# Electrospun Polymer Fiber Mats for Persulfide Prodrug Delivery

**DOI:** 10.1021/acs.biomac.5c00975

**Published:** 2025-08-01

**Authors:** Sarah N. Swilley, Hao Wu, Clarissa Tomasina, Lorenzo Moroni, Paul Wieringa, Matthew B. Baker, John B. Matson

**Affiliations:** † MERLN Institute for Technology-Inspired Regenerative Medicine, Complex Tissue Regeneration Department, 5211 Maastricht University, P.O. Box 616, Maastricht 6200MD, The Netherlands; ‡ Department of Chemistry, 1757 Virginia Tech Center for Drug Discovery, Macromolecules Innovation Institute, 1040 Drillfield Dr, Blacksburg, Virginia 24061, United States; § MERLN Institute for Technology-Inspired Regenerative Medicine, Instructive Biomaterials Engineering Department, Maastricht University, P.O. Box 616, Maastricht 6200MD, The Netherlands

## Abstract

Persulfides (RSSH) and hydrogen sulfide (H_2_S) are endogenously produced bioactive molecules that serve important roles in vasculogenesis and tissue repair processes. Through the physical incorporation of RSSH/H_2_S donors into electrospun fibers, we designed polymeric fiber mats that released provasculogenic and cytoprotective reactive sulfur species over biologically relevant time scales. The release of small molecules from the fiber mats showed that 20–50% of the analytes were released from the scaffolds after 4 h. Tube formation assays indicated that the persulfide donors improved tube formation in human microvascular endothelial cells (HMVECs) over baseline levels under oxidative conditions (H_2_O_2_). Analysis of HMVECs on the electrospun fiber mats demonstrated cell viability >80% for all groups tested. This design enables controlled release of small-molecule prodrugs as a novel method toward the formation of tissue engineering constructs that deliver reactive sulfur species, including RSSH, addressing an ongoing challenge in the field of tissue engineering.

## Graphical abstract

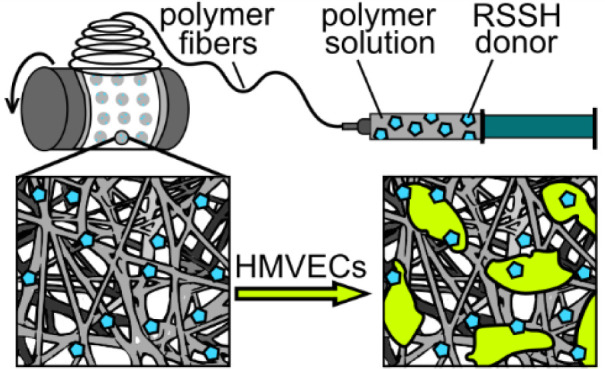

## Introduction

Reactive sulfur species, including hydrogen sulfide (H_2_S) and the related small molecule and protein persulfides (structure R–SSH), have garnered attention as potent signaling molecules due to their many biochemical roles.^1–5^ For example, H_2_S protects endothelial cells,^6,7^ cardiomyoblasts,^8^ and various other cell types^9–15^ from oxidative stress induced by reactive oxygen species (ROS), promotes vascular endothelial cell proliferation,^16,17^ and stimulates angiogenesis.^18–22^ From a tissue engineering standpoint, introducing exogenous reactive sulfur species holds potential advantages over other alternative bioactive molecules to protect cells, encourage proliferation, and promote angiogenesis. For example, while materials that release biologics can effectively promote tissue repair,^23–27^ they require the use of delicate and costly growth factors such as vascular endothelial growth factor (VEGF).^23^ As an alternative, fiber mats that release reactive sulfur species, including H_2_S and RSSH, may be more easily produced and stored as compared to VEGF. Further, it is difficult to target an effective concentration of VEGF while avoiding concentrations that can induce abnormal vasculature development.^28^ Taken together, fabricating a polymeric fiber mat for the controlled release of reactive sulfur species for use in vasculogenesis applications could enhance the effectiveness of tissue engineering constructs and reduce the reliance on VEGF.

Of the various biologically relevant reactive sulfur species, RSSH have recently gained attention as they may in fact be the primary sulfur-based signaling molecule rather than H_2_S.^29,30^ H_2_S cannot directly oxidize protein thiols. Instead, Dick and coworkers suggest that intermediate species—specifically RSSH and polysulfides formed *in vivo*—are responsible for this transformation, and the termination of the polysulfide-based signaling pathway is the reaction that produces H_2_S.^30^ However, small-molecule RSSH species are challenging to study *in vivo* and *in vitro* because they are inherently unstable in solution: Cys–SSH exhibits a half-life of 35 min at pH 7.4 in buffer and is likely much less stable in serum.^31,32^

To study RSSH species, donor compounds are needed. While Cys–SSS–Cys has been used, trisulfides are chemically unstable and not well-suited for long-term storage within polymeric fiber mats. Instead, donors in the form of stable prodrugs have emerged as key chemical tools to elucidate the biological functions of RSSH.^32,33^ Our group has created RSSH donors based on disulfides that are triggered by ROS, superoxides, esterases, and nitroreductases, with each showing the capacity to change or preserve cellular behavior in a similar fashion to H_2_S and at similar or lower levels.^34–37^ Other reports include RSSH donors that are triggered via photocleavable groups,^38,39^ biological nucleophiles,^40^ esterases,^41^ and peroxides.^42^ In summary, RSSH donors have cytoprotective abilities similar to that of many H_2_S donors.

Despite significant progress in the field of RSSH donors, very few systems have been designed for localized delivery, which is critical for tissue engineering. The high surface area-to-volume ratio of polymer fiber mats often allows for medium-term small molecule delivery,^43^ and implantable fiber mats have the unique advantage of directly delivering relevant biological factors to cells in 3D culture or to one specific location in the body.^44^ Electrospun fiber mats hold particular promise due to the many factors that can be controlled to tune their properties, including mechanical properties, porosity, surface energy, and many others.^45,46^ By varying voltage, working distance, and other parameters, electrospinning can produce fibers in the size range of native collagen fibrils^47^ with mechanical properties that enable material handling and support cell adhesion and spreading.^48^

In our previous work, electrospun poly­(caprolactone) (PCL) fiber mats functionalized with *N*-thiocarboxyanhydride H_2_S donors promoted human umbilical vein endothelial cell (HUVEC) proliferation and neovascularization *in vitro* and *in ovo*, respectively.^49^ In related work, Wang and coworkers prepared electrospun PCL fibers with another type of sequestered H_2_S donor that protected H9c2 and NIH 3T3 cells from ROS.^50^ Building on these previous examples, we set out to investigate the ability of RSSH donors to protect cells from ROS and promote viability on a fiber mat, composed of poly­(ethylene oxide terephthalate)/poly­(butylene terephthalate) (PEOT/PBT), in human microvascular endothelial cells (HMVECs). We hypothesized that physically incorporating RSSH donors (Figure 1) into an electrospun fiber mat would provide a straightforward fabrication technique, rescue HMVECs from the deleterious effects of ROS, and promote cell viability.

**Figure 1:**
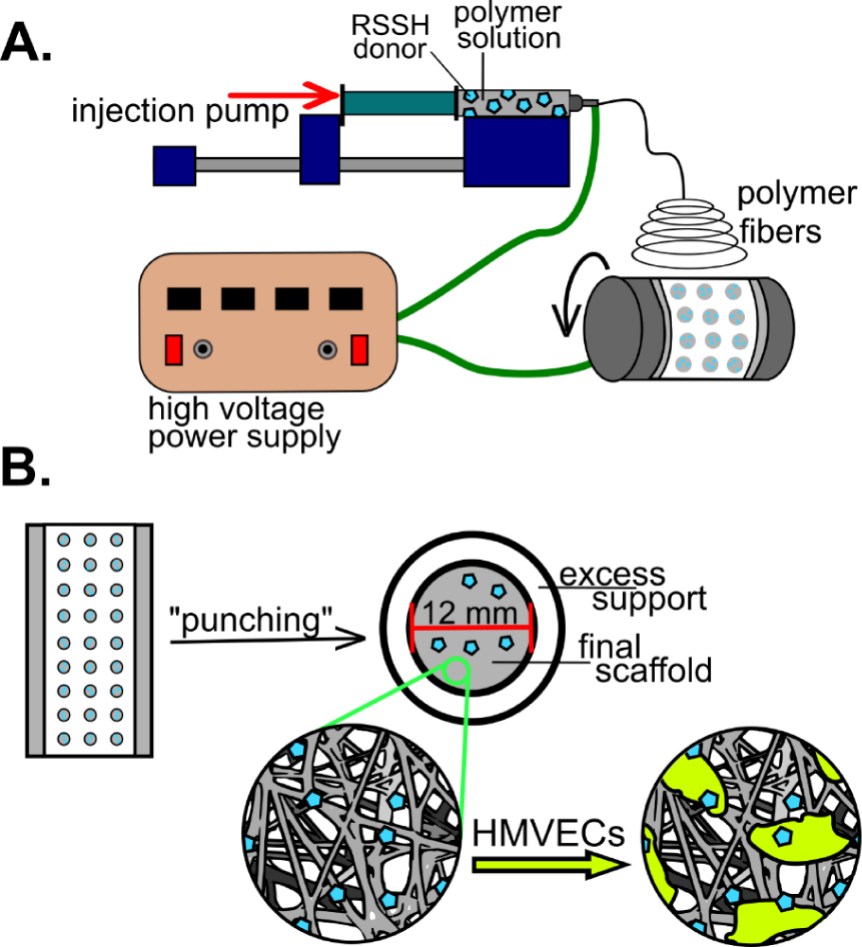
(A) Schematic illustration showing the process for electrospinning polymer fiber mats. (B) Schematic illustration of the production of final electrospun fiber mats as well as the seeding of HMVECs onto the fiber mats.

## Experimental Section

### Synthetic Procedures

**BDP-NAC**, **BDP-TE**, and **Benz-NAC** were synthesized as previously reported, and all NMR spectra matched literature precedents.^34,35^

### Electrospun Fiber Mat Fabrication

Electrospinning was performed according to a modified procedure.^51^ A solution of 18% w/v of PEOT/PBT (55:45 wt %) was prepared in a mixture of 70:30 vol % of CHCl_3_:HFIP and stirred overnight at RT. For example, 720 mg of PEOT/PBT was dissolved in 2.6 mL of anhydrous CHCl_3_ and 1.2 mL of HFIP. Separately, 5 mg of **BDP-NAC** (1.42 mmol) was dissolved in 1 mL of CHCl_3_. Next, 227.4 μL of the **BDP-NAC** solution (1.14 mg, 4 μmol) was added to the PEOT/PBT solution so that the final concentration of the drug in the spinning solution was 1 mM, and the solution was allowed to stir for 1 h prior to electrospinning.

To produce the fiber mats, first, polypropylene sheets were punched to create a series of 12 mm holes along the entirety of the sheet. Aluminum foil was first mounted onto the mandrel with double-sided tape, followed by the polypropylene sheet (Figure S1). The extrusion rate from the 0.8 mm spinneret (which was moved continuously along the length of the mandrel during the spinning process) was set to 0.5 mL/h, with a working distance of 10 cm from the mandrel. A voltage of 15 kV was applied between the spinneret and grounded mandrel (20 cm) rotating at 150 rpm. A constant temperature (25 °C) and a constant humidity (35%) were maintained throughout the entire fabrication process. After 30 min of electrospinning, the foil and polypropylene sheets were removed together, and the fiber mats were allowed to dry overnight. The following day, a 15 mm circle was punched around each fiber mat to produce the final polymer fiber mat supported by a polypropylene ring. In order to remove the aluminum foil backing for further analysis, each fiber mat was quickly dipped in 70% EtOH in H_2_O for ∼1 s before being peeled from the foil. The fiber mats were then left to dry on a flat surface overnight.

### Scanning Electron Microscopy (SEM)

SEM images were acquired using a JEOL JSM-IT200 InTouchScope SEM (JEOL, Peabody, MA, USA) operating at 10 keV. Prior to imaging, each sample was mounted onto an aluminum pin stub with carbon tape before being gold-coated with a Cressington 108 Auto Sputter Coater. Fiber dimensions were measured using an in-house-developed ImageJ macro to semi-automate manual fiber measurements. Six images at 5000× magnification were acquired for each fiber mat (Figure S2-6), and 25 fibers in each image were analyzed (*n* = 150) via Fiji software.

### Contact Angle

Surface hydrophilicity was examined via the sessile drop method with a DSA25 (KRÜSS GmbH) angle goniometer equipped with an electronic syringe unit (OCA15, Dataphysics, Germany). For this analysis, the electrospun fiber mats were laid on top of a glass slide and carefully flattened. Next, 2 μL of DI water was dropped on the surface at RT, and this process was captured via video. Images (Figure S8A) were taken from the first frame of the video in which the water droplet contacts the fiber mat’s surface.

### Differential Scanning Calorimetry (DSC)

A TA Instruments DSC 2500 was used to evaluate thermal properties of selected samples with a 10 °C/min heating and cooling ramp; data reported are from the second heat cycle (Figure S8B).

### Fourier Transform Infrared Spectroscopy (FTIR)

Each polymer fiber mat was characterized by attenuated total reflectance-Fourier transform infrared (ATR-FTIR) spectroscopy with a Nicolet iS50. Spectra were collected at RT from 400 to 4000 cm^–1^ with 32 scans (Figure S9).

### Film Thickness

Film thickness was measured with a Vectra-Touch instrument (Trimos) for mechanical test analysis. Film thicknesses were as follows: **GYY4137**-doped PEOT/PBT 138 μm, PEOT/PBT 164 μm, **Benz-NAC**-doped PEOT/PBT 120 μm, **BDP-TE**-doped PEOT/PBT 137 μm, and **BDP-NAC**-doped PEOT/PBT 169 μm.

### Mechanical Analysis

Mechanical characterization was performed with an ElectroForce 3200 instrument (TA Instruments) equipped with a 10 N load cell and operated via Win7 software. In order to prepare a fiber mat for mechanical analysis, some of the excess polypropylene support was first removed to form a rectangular shape (Figure S10). Next, samples were mounted by attaching paper strips to both sides of each polymer film with Loctite 431 adhesive glue; specifically, the polymer films were sandwiched between two pieces of paper on each edge. The samples’ dimensions were then measured with calipers before being inserted into the instrument’s gripping equipment such that only the polymer film was present between the two grips. Each sample was analyzed at a strain rate of 1% strain/s at RT. The samples were tensed until a maximum load. Ultimate tensile strength was determined via the highest stress point before sample creep from the stress–strain curve (Figure S11). Young’s modulus was calculated from the elastic region (0.02–0.1 strain) of the stress–strain curves (Figure S12).

### Release of Small Molecules from Electrospun Fiber Mats

To analyze drug release over time from the fiber mats, we utilized ^1^H NMR spectroscopy with a stock solution of 12.3 mM maleic acid (1.428 mg/mL) in 50 mM phosphate-buffered D_2_O (pD = 7.8 and pH = 7.4, adjusted with D_3_PO_4_) as the solvent. In short, 1 mL of the D_2_O stock solution was added to a scintillation vial, and two electrospun fiber mats were subsequently added. At each predetermined time point, 700 μL of the release solution was removed and added to an NMR tube for analysis, followed by adding 700 μL of fresh buffer back to the scintillation vial containing the films. Each aliquot was analyzed via ^1^H NMR spectroscopy by first integrating the maleic acid peaks at 5.91 ppm to 1000 before integrating the signal at 1.84 ppm or at 3.26 ppm in the case of **GYY4137**. The relative protons in each structure are highlighted in red in Figure 2B.

### Cell Culture

Cell studies were performed using human microvascular endothelial cells (HMVECs, CC-2516, Lonza) at passage 4-8. HMVECs were cultured in an endothelial cell growth medium (EGM-MV2, C-22022, PromoCell). Cultures were incubated under a humidified environment with 5% CO_2_ at 37 °C. The culture medium was changed every other day, and cultures were passaged at 80% confluence to prevent contact inhibition. Cells were washed with 1× PBS and then released with 0.05% trypsin-EDTA solution. The suspension of released cells was centrifuged at 200 × *g* (1000 rpm) for 5 min before counting and plating for the experiments.

### Cell Viability after 1 h of Exposure to Small Molecules

HMVECs at passages 4–8 were utilized for all cell studies. HMVECs were seeded in 96-well plates at a seeding density of 10k cells/well. The cells were cultured for 24 h, after which the medium was discarded, and the cells were washed three times with 1× PBS. Stock solutions of **GYY4137** (20 mM), **BDP-NAC** (200 mM), **BDP-TE** (200 mM), and **Benz-NAC** (200 mM) were prepared by dissolving the desired amounts of the chemicals in DMSO. Next, varying concentrations of **GYY4137** (100 μM to 200 μM), **BDP-NAC** (50 μM to 200 μM), **BDP-TE** (50 μM to 200 μM), and **Benz-NAC** (50 μM to 200 μM) were prepared from each DMSO stock solution into EGM-MV2. Na_2_S (100 μM to 200 μM) was prepared by dissolving the desired amount directly into EGM-MV2. For the solvent group, DMSO was diluted 100 times in EGM-MV2. To make the stock solution of H_2_O_2_, 30% H_2_O_2_ (9.8 M) was used to make a 5 mM H_2_O_2_ solution in EGM-MV2, and 2 μL was added to each well to get a final H_2_O_2_ concentration of 100 μM. To each well was added 100 μL of each solution, followed immediately by H_2_O_2_ (100 μM), and the cells were incubated for 1 h. After 1 h, the media was discarded, and the cells were washed three times with 1× PBS. Finally, the cells were cultured with EGM-MV2 for either 1 day or 3 days, upon which three assays were performed: live/dead staining, PrestoBlue, and a DNA assay.

### Live/Dead Staining

Cell cytotoxicity was assessed using live/dead staining, which included three components: Hoechst (nucleic acid labeling dye), calcein AM (live-cell labeling dye), and propidium iodide (PI, dead-cell labeling dye), to simultaneously determine the existence of live and dead cells. After 1 or 3 days of culture post exposure to the small molecule analytes as described above, the cells were washed with PBS once and incubated in 5 μg/mL Hoechst (staining nuclei), 1 μM calcein AM (staining live cells), and 1 μg/mL PI (staining dead cells) in PBS for 20 min at 37 °C. The cells were then washed with PBS three times to remove excess dye. Finally, the samples were observed under a fluorescent microscope (Figures S14 and S15).

### PrestoBlue

Cell viability was analyzed using the PrestoBlue assay according to the manufacturer’s protocol (Fisher Scientific). The PrestoBlue reagent (10% V/V) was mixed with EGM-MV2 to prepare the PrestoBlue medium. After 1 or 3 days of culture post exposure to the small molecules as described above, 100 μL of the PrestoBlue medium was added to each well and then incubated at 37 °C for 30 min. Next, the media from each well was transferred into a black 96-well plate with a clear bottom. The fluorescence emission was measured at 540–570 nm excitation and 580–610 nm emission in a microplate reader (CLARIOstar, BMG LABTECH). The readout from the samples was corrected with a control (PrestoBlue medium) (Figures 6 and S16).

### DNA Assay

The DNA concentration, based on the total amount of DNA of each sample, was quantitatively determined with the CyQUANT Cell Proliferation Assay Kit (Thermo Fisher Scientific) at 1 and 3 days after the 1 h analyte and H_2_O_2_ treatment. The cells were first cycled through freeze-thaw three times (30 min at −80 °C and 30 min at RT). Then, they were digested overnight with 150 μL of Proteinase K in a Tris/EDTA solution at 56 °C. The CyQUANT GR dye and lysis buffer were prepared according to the manufacturer’s protocol, adding 150 μL of lysis buffer containing RNase A (1:500) to each well and left at RT for 1 h to degrade the cellular RNA. Next, 100 μL of the digested samples was transferred to a black 96-well plate, and the GR dye solution (100 μL) was added to each well. After incubating the samples at room temperature for 15 min, the fluorescence intensity of the samples was measured using a microplate reader (Figures 6 and S16).

### Live/Dead Staining after 16 h Exposure to Varying Concentrations of H_2_O_2_

HMVECs were seeded in 96-well plates at a seeding density of 10k cells/well. The cells were cultured for 24 h, after which the medium was discarded, and the cells were washed three times with 1× PBS. EGM-MV2 was added to the seeded cells, and H_2_O_2_ (100 μL total in each well) was added to each well before incubating the cells for 16 h. 30% H_2_O_2_ (9.8 M) was used to make a 5 mM H_2_O_2_ solution in EGM-MV2. Next, varying concentrations of H_2_O_2_ (100 to 1000 μM) were prepared from the 5 mM H_2_O_2_ solution in EGM-MV2. The media was discarded after 16 h, and the cells were washed three times with 1× PBS. Then, live/dead staining was performed as described above. The live cell proportion was assessed by counting the number of calcein AM-stained viable cells and PI-stained dead cells using NIS analysis software (Figure S17).

### Live/Dead Staining after 16 h Exposure to 600 or 800 μM H_2_O_2_ and/or 200 μM of Na_2_S, or BDP-NAC

HMVECs were seeded in 96-well plates at a seeding density of 10k cells/well. The cells were cultured for 24 h, after which the media was discarded, and the cells were washed three times with 1× PBS. Next, 100 μL of EGM-MV2 was added to the seeded cells, followed by 200 μM Na_2_S or 200 μM **BDP-NAC**. Finally, either 600 or 800 μM H_2_O_2_ was added, and the cells were incubated for 16 h.

To make the stock solution of H_2_O_2_, 30% H_2_O_2_ (9.8 M) was used to make a 5 mM H_2_O_2_ solution in EGM-MV2, and 12 or 16 μL was added to each well to achieve a final H_2_O_2_ concentration of 600 or 800 μM. A stock solution of **BDP-NAC** (200 mM) was prepared by dissolving the chemical in DMSO. Next, **BDP-NAC** (200 μM) was prepared from the stock solution in DMSO by diluting into EGM-MV2. Na_2_S (200 μM) was prepared by dissolving the desired amount in EGM-MV2 directly.

After 16 h, the medium was discarded, and the cells were washed three times with 1× PBS. Then, live/dead staining was performed as described above. The percentage of live cells was assessed by counting the number of calcein AM-stained viable cells and PI-stained dead cells using NIS (Nikon Imaging Software) analysis software (Figures S18 and S19).

### Angiogenesis Assay after 16 h Exposure to 600 μM H_2_O_2_ and/or 200 μM of Na_2_S, or BDP-NAC Both with and without VEGF Present in the Growth Medium

First, 150 μL of Geltrex was added to a 48-well plate. The plate was subsequently incubated at 37 °C and 95% humidity for 30 min to cross-link the gel. Next, HMVECs were seeded on top of the gel at a seeding density of 20k cells/well within 220 μL of EGM-MV2 (containing 0.5 ng/mL VEGF165, or, for the study without VEGF165, this step was excluded), containing 200 μM Na_2_S or **BDP-NAC**. Lastly, 30 μL of H_2_O_2_ (5 mM) was added to each well to reach a final H_2_O_2_ concentration of 600 μM. The cells were incubated for 16 h, upon which bright-field images were acquired. Each group selected at least eight fields of view for imaging. Fiji (Plugin: Angiogenesis Analyzer) was used for analyzing the angiogenesis results of each group (Figures 8, 9, and S20). This assay was also performed identically as described above, but without the addition of VEGF165 (Figures S21 and S22).

### Cell Culture on Electrospun Fiber Mats

To prepare the fiber mats for cell culture, the mats were quickly dipped into 70% EtOH in H_2_O to detach the fiber mat from the aluminum foil backing. Next, the fiber mats were sterilized by incubating them in a CX-2000 UV cross-linker at 254 nm for 15 min. The fiber mats were then added to a 24-well plate before adding an O-ring (Eriks) on top of the fiber mat to prevent floating during cell culture. The fiber mats were washed three times with 1× PBS and once with EGM-MV2 before seeding HMVECs at a density of 120k cells/well within 300 μL of EGM-MV2. Assays including H_2_O_2_ were performed by immediately adding H_2_O_2_ to the wells to reach a final concentration of 600 μM after the addition of cells. The cells were incubated for 16 h, upon which they were washed three times with 1× PBS before performing live/dead staining as described above. After incubating the cells with the dyes, the scaffolds were flipped 180° and transferred to a glass-bottom Petri dish for imaging (Figures 10 and S24).

### Quantification of Cell Viability on Electrospun Fiber Mats

Cell viability on the electrospun scaffolds was determined as follows: first, individual channels from Figure S24 were opened in ImageJ, and using the Otsu function in the color thresholding tool, the images were adjusted such that only the cells were highlighted within the software. Next, the measurement tool was utilized to measure the number of cells in each image, specifically using the “counts” data that was generated. The % error between the actual total counts to determine live/dead (green/(red+green)) and the theoretical maximum number of counts via the blue channel was determined; the blue channel was the best resolved, as can be seen in Figure S24. If the error between these two measurements was <20%, then these calculated values were utilized to generate the data in Figure S25. If the error between these two values was >20%, the number of cells in the green and red channels was calculated manually, and this yielded values <20% error despite manually counting the cells. However, one of the five images for both **Benz-NAC** and PEOT/PBT + H_2_O_2_ yielded errors >20%, and therefore these two data sets in Figure S25 only consist of four measurements each. This variability between the ImageJ software counting the numbers of cells in each channel was attributed to poor image quality in the red and green channels as a result of imaging the cells in a 2.5D matrix, which caused some blurriness among the images; this accounts for the need to manually count some images.

## Results and Discussion

In choosing a fiber mat for electrospinning, we set out to select a material that would undergo degradation in the body, could be easily electrospun, and would support cell growth. Ultimately, we chose a copolymer of PEOT/PBT, commercially available as Polyactive (Figure 2A) 300 PEOT/PBT 55:45, where 300 refers to the initial *M*_w_ of PEG used in the copolymerization reaction and 55:45 to the weight ratio between PEOT and PBT. PEOT/PBT has been electrospun for applications in bone tissue regeneration,^52^ localized cancer therapy,^53^ enhanced cell proliferation,^54^ and anti-inflammatory applications.^55^

**Figure 2:**
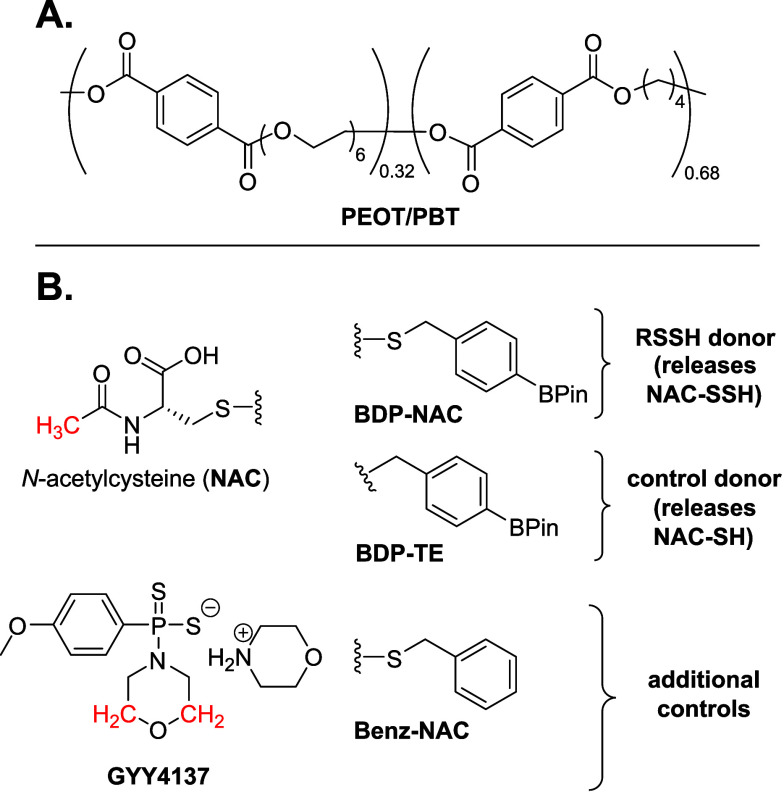
(A) Chemical structure of PEOT/PBT and (B) structures of small molecule RSSH donors utilized in this study as well as GYY4137, a slow-releasing H_2_S donor. Pertinent protons for ^1^H NMR analysis utilized for small molecule release studies are highlighted in red.

Based on the hypothesis that RSSH donors could protect HMVECs from oxidative stress, both in solution and when released from electrospun fiber mats, we designed this study to include an RSSH donor that responded to ROS along with relevant control compounds (Figure 2B RSSH donors). The RSSH donor **BDP-NAC** was selected based on its reactivity toward biological triggers, specifically oxidizing metabolites (H_2_O_2_). **BDP-NAC** contains a pinacol boronic ester (BPin) unit that responds to H_2_O_2_ to form a phenoxide, leading to 1,6-elimination (Scheme S1A). We envisioned that H_2_O_2_-triggered release would be useful in environments where oxidative stress is high, for example, after myocardial infarction^56^ or during wound healing.^57^

Three critical control compounds were also included: (1) **BDP-TE**, where “TE” indicates a thioether (Figure 2B control donor). **BDP-TE** is also triggered by H_2_O_2_ but releases NAC–SH rather than NAC–SSH due to the thioether bond instead of a disulfide (Scheme S1B). Therefore, it serves as a control compound that simply lacks one sulfur atom compared to the active small molecule **BDP-NAC**. (2) **Benz-NAC**, which has no triggering unit on the aromatic ring and therefore cannot release NAC–SSH but is otherwise structurally similar to **BDP-NAC**. (3) Finally, we also included **GYY4137** (Figure 2B additional control), a commercially available slow-releasing H_2_S donor,^58^ in the studies to serve as an H_2_S donor, allowing us to compare the effects of RSSH vs H_2_S in cell studies.

### Fiber Mat Preparation and Characterization

Each polymer solution used for electrospinning started from a solution of PEOT/PBT in a mixture of CHCl_3_ and hexafluoroisopropanol (HFIP). To this solution was added each corresponding small molecule at a final concentration of 1 mM. Each polymer/small molecule solution was electrospun under standardized conditions (Figures 1 and S1). The fiber mats were then dried before characterization and release studies. Upon completion of electrospinning of the different material compositions, i.e., PEOT/PBT alone or PEOT/PBT doped with small molecules, we first characterized the polymer fibers via SEM imaging. Six images were taken from different sections of the polymer fiber mats at varying magnifications (Figures S2–S6). As shown in Figure 3A–D, each of the five groups examined exhibited a random fiber morphology.

**Figure 3:**
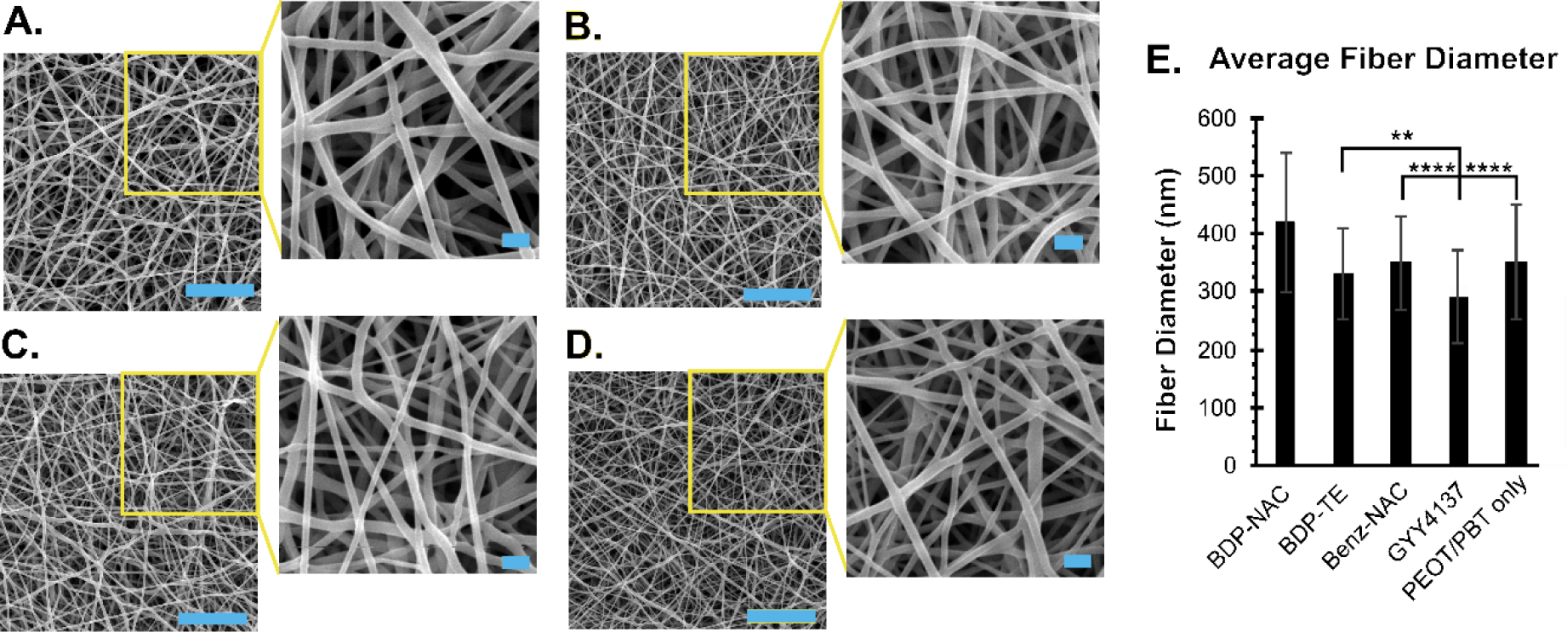
Scanning electron microscopy images of selected electrospun polymer fibers with or without small molecules. (A) **BDP-NAC**-doped PEOT/PBT, (B) **BDP-TE**-doped PEOT/PBT, (C) **GYY4137**-doped PEOT/PBT, and (D) PEOT/PBT without small molecule; scale bar is 5 μm, and the scale bar for the inset images is 1 μm. (E) Summary of fiber diameters. Error bars indicate the standard deviation of 150 fiber measurements across six different images for each sample. Individual histograms and statistical analysis between each group can be seen in Figure S7. Statistical analyses were performed using one-way ANOVA followed by Tukey post hoc tests. BDP-NAC films were found to have a significance of *p* < 0.0001 between all groups. **** indicates *p* < 0.0001, *** indicates *p* < 0.001, ** indicates *p* < 0.01, and * indicates *p* < 0.05.

The fiber mats doped with small molecules exhibited average fiber diameters between 280–420 nm, while PEOT/PBT fibers alone exhibited an average fiber diameter of 340 nm (Figure 3E). The diameter of these fibers is on the same size scale as collagen fibrils.^59^ We counted 150 fibers for each group, and while some diameters were statistically different between groups, there were no clear trends in diameter, and all were in a similar range. Although statistical differences were noted, the overall range of the average diameters is fairly narrow, and we did not expect that these small differences in fiber diameters would affect further studies of the electrospun fiber mats.

After each material formulation was electrospun, we investigated the surface wettability of the fiber mats. After flattening a fiber mat onto a glass slide, a water droplet was deposited onto the fiber mat surface, and in all cases, the droplets instantly penetrated the fiber mats (Figure S8). Because the water spread quickly across the fiber mat surface over a period of seconds, it was not possible to obtain accurate quantitative measurements of the contact angles. As such, this rapid spreading of water across the fiber mats was taken to indicate that the fiber mats were all of similar hydrophilicity with good surface wettability, as reported by similar studies using PEOT/PBT as the main scaffold material.^52^

Next, we analyzed the physical properties of the polymer fiber mats via tensile testing. Tensile tests were run at a rate of 1% strain per second at RT. Each fiber mat was analyzed in triplicate, where individual analyses can be seen in Figures S11 and S12, and representative stress–strain curves are shown in Figure 4. Interestingly, statistical analysis (Figure S11F) indicated that there were no statistically significant differences in the strain at failure between any of the groups examined, with all in the range of 40–55% strain at failure.

**Figure 4:**
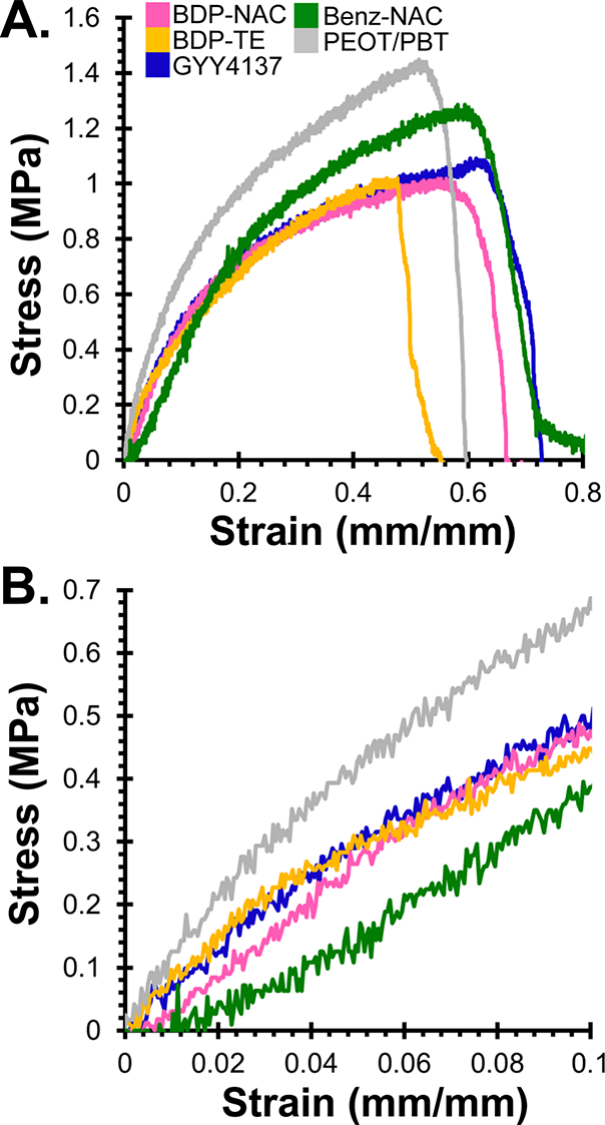
(A) Representative stress–strain curves of the fiber mats. (B) Inset from panel (A) to highlight the initial slope in the elastic region. Individual analyses with statistical tests can be seen in Figures S11 and S12.

Interestingly, there were small differences in the ultimate tensile strength of the different fiber mats. From Figure 4A it is clear that PEOT/PBT (no additive) had the highest ultimate tensile strength at 1.4 ± 0.1 MPa. Again, the other materials tested exhibited lower ultimate tensile strength as compared with the PEOT/PBT fiber mats, with values ranging from 0.9 to 1.3 MPa. This trend, in which most of the doped polymers exhibited somewhat lower ultimate tensile strength than PEOT/PBT, could be attributed to the small molecules incorporated within the PEOT/PBT acting as plasticizers, a phenomenon that has been reported previously.^60^

In order to determine the Young’s modulus of each fiber mat, we analyzed the linear regions of the stress-strain curves (Figures 4B and S12), and the results are summarized in Table 1. We found that pristine PEOT/PBT fibers exhibited the highest Young’s modulus among the groups tested, with a value of 5.7 MPa. Other fiber mats exhibited Young’s moduli between 3.8 and 5.4 MPa, which are similar to that of PEOT/PBT. Again, it is typical for small molecules to act as plasticizers in PEOT/PBT, lowering the Young’s modulus somewhat compared to pristine PEOT/PBT. Because the overall range in ultimate tensile strength and Young’s modulus was fairly small (<2-fold difference between the highest and lowest values), we concluded that the fiber mats had similar enough mechanical properties to make reasonable comparisons among them in the cell culture studies.

**Table 1:** Summary of the Mechanical Properties of Doped PEOT/PBT Fiber Mats

	Strain at Failure (%)^a^	Ultimate Tensile Strength (MPa)^a^	Young’s Modulus (MPa)^b^
BDP-NAC	47 ± 8	1.0 ± 0.1	5.0 ± 0.5
BDP-TE	50 ± 10	0.9 ± 0.1	3.8 ± 0.9
Benz-NAC	50 ± 4	1.0 ± 0.2	4.2 ± 0.4
GYY4137	57 ± 7	1.3 ± 0.2	5.4 ± 1.5
PEOT/PBT	51 ± 3	1.4 ± 0.1	5.7 ± 0.2

a Strain at failure and ultimate tensile strength were determined from the highest stress point before sample creep.

b Young’s modulus was determined from the linear regions highlighted in Figures S12 and 4B. For strain at failure, no statistical significance was found between any of the groups examined (Figure S11F). Other statistical analyses can be found in the Supporting Information: ultimate tensile strength (Figure S11G) and Young’s modulus (Figure S12F).

### Small Molecule Release from Electrospun Fiber Mats

To analyze small molecule release over time from each of the scaffolds, we first attempted to follow the release using UV–vis analysis. Unfortunately, commercially supplied PEOT/PBT contains tocopherol (a derivative of vitamin D), and tocopherol has a characteristic absorption peak at 230 nm that overlaps with the major absorption peak from all of the NAC-based donors. Instead, we utilized ^1^H NMR spectroscopy in D_2_O with maleic acid as an internal standard to follow the release. Fiber mats were immersed in 50 mM phosphate-buffered D_2_O containing a known concentration of maleic acid, and at each time point, the release solution was transferred to an NMR tube for analysis. The aliquot volume was then replaced with fresh D_2_O buffer. Each aliquot was analyzed via ^1^H NMR spectroscopy, measuring the integral of the signals corresponding to the protons highlighted in Figure 2B. Release results are displayed in Figure 5.

**Figure 5:**
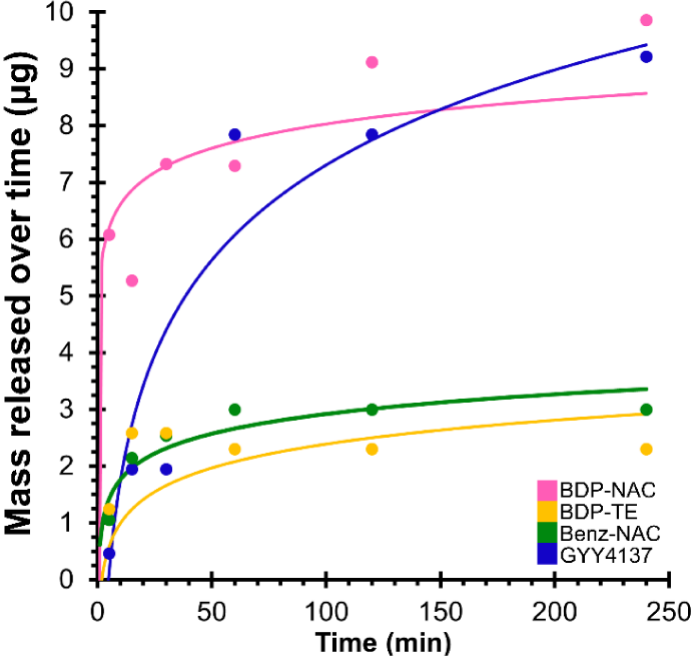
Representative small molecule release from different electrospun fiber mats (Figure S13). Experiments were conducted in a stock solution of 12.3 mM maleic acid in 50 mM phosphate-buffered D_2_O (pD = 7.8, equivalent to pH = 7.4, adjusted with D_3_PO_4_) as the solvent. The solid lines indicate a logarithmic fit to the experimental data points. The small molecule loading for each compound in this series of experiments was similar, around 18 μg.

Analysis of the ^1^H NMR spectroscopy data allowed us to measure the mass of each small molecule released over the course of 4 h, which was sufficiently long to see differences in release while avoiding solubility problems at longer times. Across the four types of small molecules, between 2–9 μg of the analytes were released after 4 h, corresponding to 20–50% of the total payload of each fiber mat after 4 h. These results indicate that 50–80% of the small molecules were still encapsulated within the polymer fiber mats, and therefore, there is potential for more small molecules to be released after the onset of polymer degradation or further incubation in solution. We anticipate that the release kinetics of our systems in a more complex *in vitro* environment would provide trends similar to those of the buffered model study. This assumption is supported by recent work from Focarete and coworkers, who monitored the release of GYY4137 from a multilayer scaffold in buffer and validated these release studies by demonstrating protective dose-dependent effects *in vitro*.^61^

As shown in Figure 3, the morphology and diameter of each of the polymer fibers were relatively similar, as were their mechanical properties (Table 1). Therefore, we attribute the differences in release rates to differences among the chemical structures of the small molecules themselves or their potential physical interactions with the fiber matrix, rather than a function of the polymer fibers. This same effect has been reported previously by Narasimham and Langer.^65^ They found that the rapid release of small molecules from polymeric “hemispheres” largely depended on the solubility of the small molecule in solution. Similarly, other studies report that heterogeneity of the polymer matrix and small molecule solubility, among other factors, contribute to fast release profiles.^65^ We postulate that the quick release of **GYY4137** could be attributed to its excellent water solubility (30 mg/mL H_2_O at RT)^58^ and that the molecule exists in a salt form, whereas the other small molecules are neutral organic compounds. Conversely, the limited water solubility of **Benz-NAC**, for example (<1 mg/mL), could contribute to the significantly slower release rate compared to the other analytes.

The high surface area-to-volume ratio of polymer fiber mats often gives rise to a quick release of their small-molecule payloads. Additionally, polymeric delivery vehicles with physically incorporated small molecules, such as these fiber mats, also tend to have faster release rates compared to covalently attached donors.^43^ Similarly, this phenomenon was reported in PEOT/PBT systems, where dyes of varying molecular weights also exhibited burst release profiles.^54^ Therefore, we postulate that the solubility of the NAC derivatives and **GYY4137** in the release media could explain the differing release rates from those of the polymer fiber mats. We hypothesized that the prolonged release of the RSSH donors from the fiber mats would give rise to increased cell viability/proliferation compared to small-molecule assays, potentially due to longer-term exposure of the cells to the small molecules.

### Cell Studies of Small Molecule RSSH/H_2_S Donors and Controls

Before analyzing the behavior of HMVECs on the polymer fiber mats, we first performed multiple biological assays utilizing the small-molecule RSSH/H_2_S donors and control compounds in 2D cultures, as none had previously been tested on HMVECs. HUVECs are derived from umbilical veins, while HMVECs are derived from microvessels and are more representative of microvasculature formation. First, we performed live/dead staining after 1 day (Figure S14) and 3 days (Figure S15) of exposure to 100 μM H_2_O_2_ and 200 μM of each of the small molecules. As shown in Figures S14 and S15, after both 1 and 3 days, each of the groups closely resembled the control group, where all images showed mostly live cells.

To better understand the effect of the small molecules on the growth of HMVECs, we also performed a quantitative cell viability and cell proliferation study using PrestoBlue and DNA assays, respectively, again analyzing both at 1 day (Figure S16) and 3 days (Figure 6). Due to the limited water solubility of the persulfide donors, each small molecule was first dissolved in DMSO; therefore, we evaluated cell viability in the presence of DMSO as well. We note that both **BDP-NAC** and **BDP-TE** require peroxides to trigger the release of NAC-SSH or NAC-SH, respectively; to account for the addition of peroxides in the **BDP-NAC**/**BDP-TE** groups, we included H_2_O_2_ (100 μM) in all small molecule assays. Cells were exposed to H_2_O_2_ and/or the small molecules for 1 h before being washed with media. To compare cell viability between the groups, we chose to use the DMSO + H_2_O_2_ group as the control group. We determined that all small molecules tested were nontoxic up to a concentration of 200 μM. After 1 day (Figure S16A) and 3 days (Figure 6A), there were no significant differences among all groups tested. We next analyzed cell proliferation via the total DNA content after 1 day (Figure S16B) and 3 days (Figure 6B). Our results were nearly identical to those found in the PrestoBlue assay, in that no significance was found among the groups tested.

**Figure 6:**
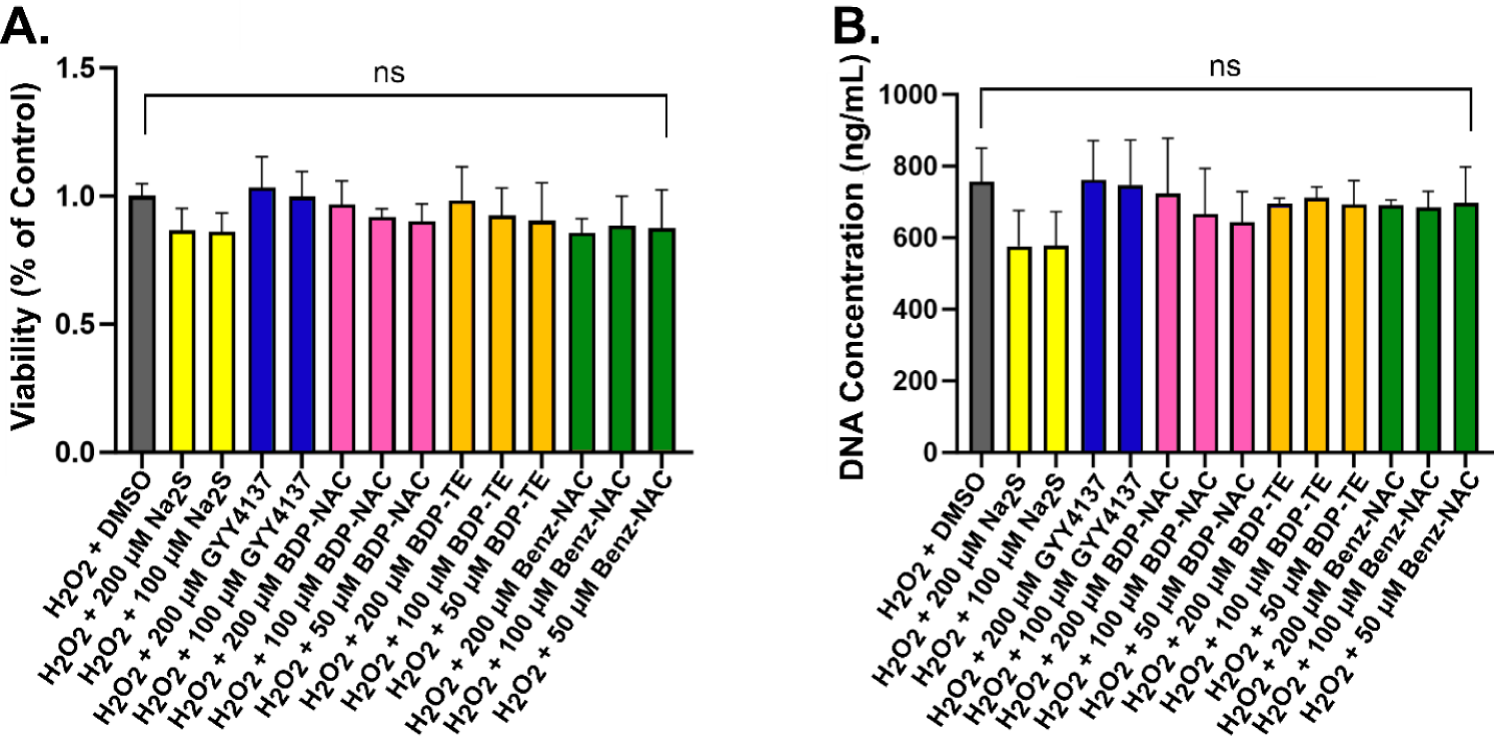
(A) PrestoBlue viability assay of HMVECs, and (B) concentration of DNA present in HMVECs after exposure to the small molecules at varying concentrations along with 100 μM H_2_O_2_ for 1 h, after 3 days of culture. Viability was normalized to the H_2_O_2_ + DMSO group. DNA concentration was determined via an Invitrogen C7026 DNA assay kit. **** indicates *p* < 0.0001, *** indicates *p* < 0.001, ** indicates *p* < 0.01, and * indicates *p* < 0.05. Error bars represent the standard deviation of the mean for three independent experiments with *n* = 3 for each experiment.

The ability of the small molecules to rescue HMVECs from H_2_O_2_ exposure was of particular interest to us because oxidative stress is common in tissue repair. We first conducted a live/dead staining assay to determine how various H_2_O_2_ concentrations affected cell viability (Figure S17). At concentrations up to 400 μM H_2_O_2_, cell viability was unaffected (Figure 7A); however, at 600 μM, only 80% of cells were viable, and at 800 μM, <5% of cells remained viable. We chose to use both 600 and 800 μM H_2_O_2_ in the subsequent assays in order to examine the effects of **BDP-NAC** on cells at concentrations of H_2_O_2_ that either minorly or significantly affect cell growth. We note here that HMVECs are significantly more robust than HUVECs; just 200 μM of H_2_O_2_ kills almost the entirety of the HUVEC cell population^62^ highlighting the need for testing in relevant cell types.

**Figure 7:**
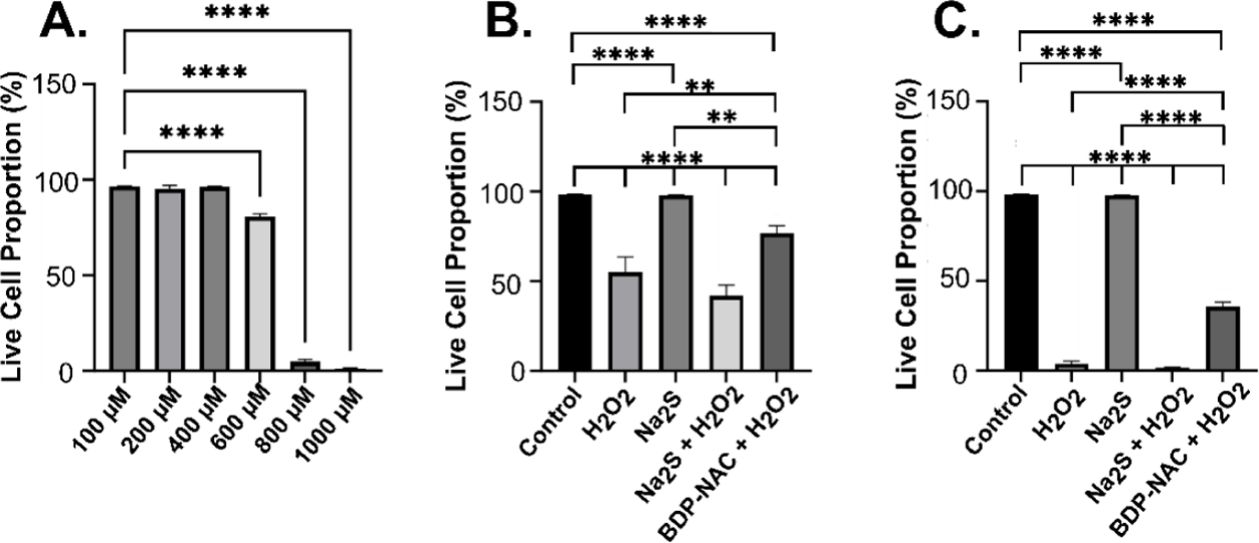
(A) Bar graph quantifying the live cell proportion, as compared to untreated cells (Control), when exposed to varying concentrations of H_2_O_2_. (B,C) Bar graphs indicating the live cell proportion of HMVECs as compared to untreated cells after exposure to (B) 600 μM H_2_O_2_ or (C) 800 μM H_2_O_2_ and/or 200 μM of the small molecules. **** indicates *p* < 0.0001, *** indicates *p* < 0.001, ** indicates *p* < 0.01, and * indicates *p* < 0.05. Error bars represent the standard deviation of the mean for three independent experiments with *n* = 3 for each experiment.

In the initial assay, utilizing passage number 6, we found that 80% of HMVECs were viable when treated with 600 μM H_2_O_2_. Using a later passage number of the HMVECs, we found that upon exposure to 600 μM H_2_O_2_ only ∼50% of HMVECs were viable in passage number 7 (Figures 7B and S18). We have found that this level of variability is common in H_2_O_2_ treatment studies, so we completed the rest of the studies with passage 7 cells (Figure 7B,C). When incubated with H_2_O_2_ and Na_2_S, cell viability decreased to 45%, while the addition of H_2_O_2_ with **BDP-NAC** increased viability to 75%, indicating the ability of **BDP-NAC** to rescue HMVECs from oxidative stress; these results are in line with the previous observations of **BDP-NAC** in H9c2 cardiomyocyte cells.^34^ Likewise, when exposed to 800 μM H_2_O_2_, nearly all of the cells were dead, but **BDP-NAC** was again able to rescue some cells, with 30% of the cells still viable (Figures 7C and S19).

Next, we utilized a tube formation assay to determine the ability of the NAC derivatives and controls to stimulate or affect the growth of HMVECs into tube-like structures. We performed this assay both with and without VEGF in order to examine if the small molecule donors could induce vasculogenesis in the absence of the growth factor. We show here (Figure 8) colorized images of HMVECs after the vasculogenesis assay without VEGF; images without the colorized guide can be seen in Figure S20, and images of this assay with VEGF can be seen in Figures S21 and S22.

**Figure 8:**
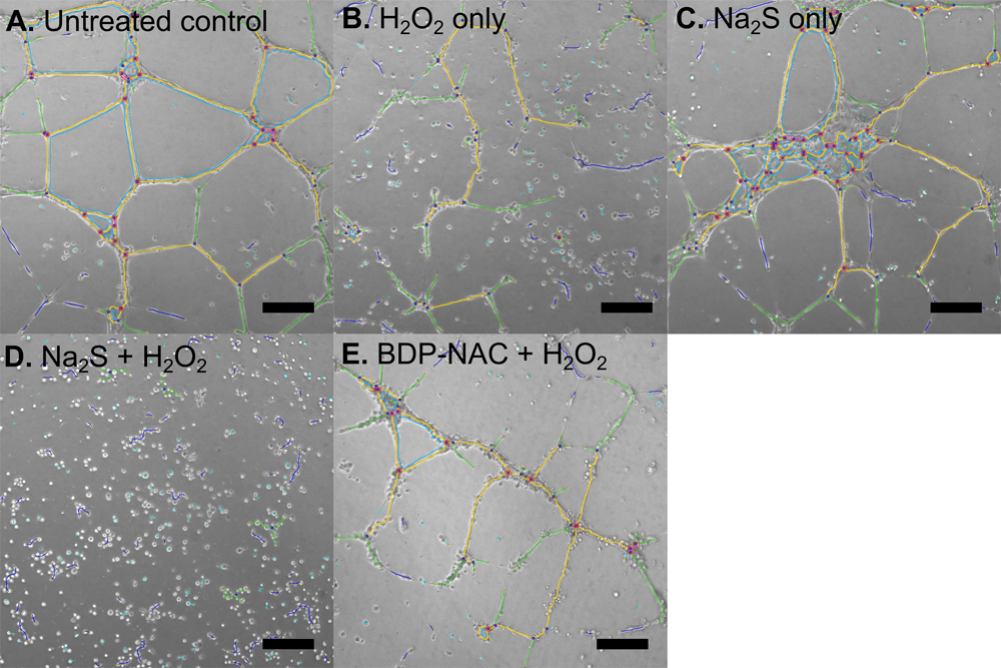
Colorized tube formation images using Fiji software after 18 h of incubation; 600 μM H_2_O_2_ and/or 200 μM of each small molecule tested in media without VEGF. (A) Control, (B) H_2_O_2_ only, (C) Na_2_S only, (D) Na_2_S + H_2_O_2_, and (E) BDP-NAC + H_2_O_2_. Scale bar = 200 μm. Extremities: red dot; junctions: blue circle; branches: green line; segments: yellow line; meshes: blue area.

The untreated control group (Figure 8A) showed well-defined formation of structures, while the group treated with H_2_O_2_ only (Figure 8B) showed a clear disruption of cell organization. Cells treated with Na_2_S only (Figure 8C) exhibited similar growth compared to the control group. Again, when exposed to both Na_2_S and H_2_O_2_ simultaneously, the cells underwent cell rounding, which can be seen clearly in Figure 8D. Finally, Figure 8E shows that coadministration of **BDP-NAC** and H_2_O_2_ restores the cells’ ability to organize into defined structures, as compared to H_2_O_2_ only (Figure 8B).

To better quantify the data from Figure 8, we summarized the results into a series of bar graphs (Figure 9). Similarly, a bar graph summarizing the results of this assay with VEGF can be seen in Figure S23; we observed similar trends between the groups with/without VEGF. Of note, the studies with added VEGF clearly showed increased vascular network formation in some metrics (e.g., the number of junctions). Because we are more interested in the ability of these donors to promote tube formation in the absence of exogenous VEGF, we discuss only the results from the assay without additional VEGF here. However, there is certainly a small amount of VEGF remaining in the fetal bovine serum that was added to the medium and the Geltrex matrix; therefore, these studies represent low levels of VEGF rather than a complete lack of VEGF.

**Figure 9:**
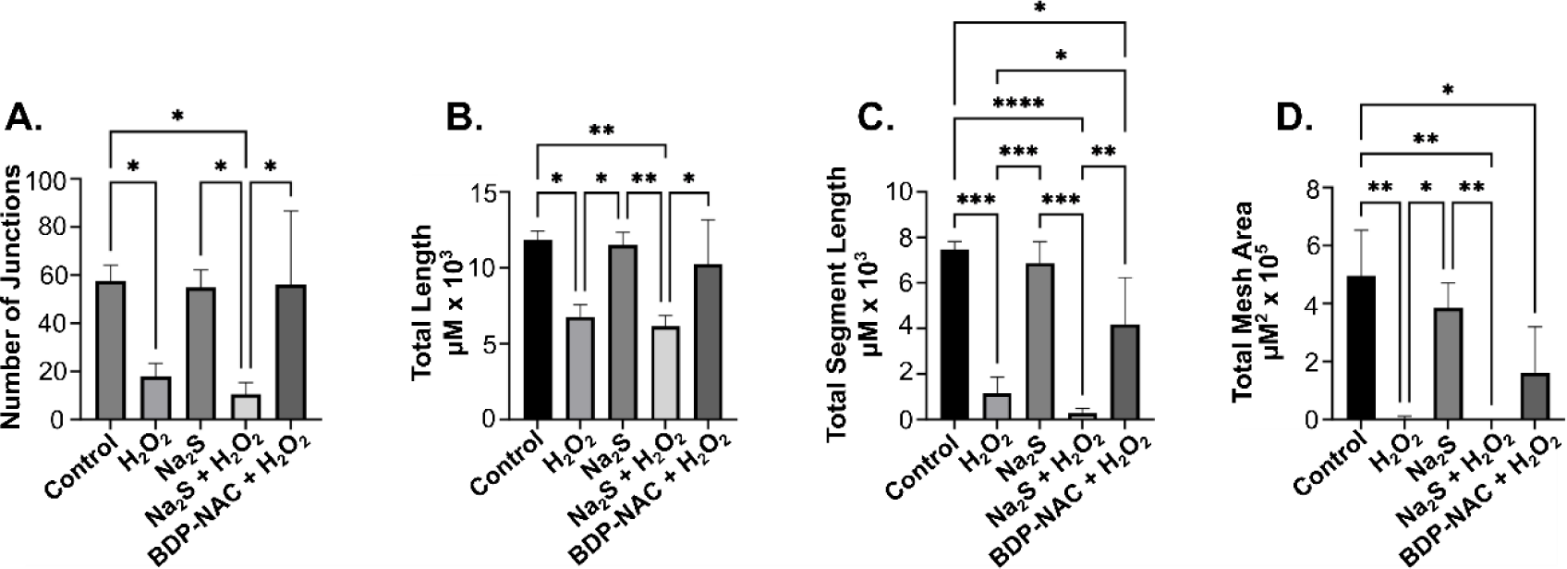
Bar graphs quantifying the (A) number of junctions, (B) total length, (C) total segment length, and (D) total mesh area of HMVECs utilizing 600 μM H_2_O_2_ and/or 200 μM of each small molecule tested in media without VEGF after 18 h. **** indicates *p* < 0.0001, *** indicates *p* < 0.001, ** indicates *p* < 0.01, and * indicates *p* < 0.05. Error bars represent the standard deviation of the mean for three independent experiments with *n* = 3 for each experiment.

In summary, exposure of HMVECs to 600 μM H_2_O_2_ decreased the number of junctions, segment length, and mesh area significantly compared to the untreated control group. Each type of measurement (Figure 9A–D) indicated that exposure to H_2_O_2_ (second treatment group) decreased cell organization by more than half compared to the control group. In the third treatment group, Na_2_S alone (no H_2_O_2_), all measurements were statistically identical with those of the control (untreated) group. Cells treated with both Na_2_S and H_2_O_2_ (fourth treatment group), however, led to drastic changes. All measurements decreased significantly from the control group, with the total segment length and total mesh area both dropping to nearly zero. When **BDP-NAC** was coadministered with H_2_O_2_ (fifth treatment group), some cellular function was restored as compared to the H_2_O_2_-only treated cells. The number of junctions, total (segment) length, and total mesh area all increased. This effect is, presumably, due to the RSSH released from **BDP-NAC** protecting the cells from oxidative stress.^34^

### Cell Culture on Electrospun Fiber Mats

Finally, we investigated cell proliferation on electrospun fiber mats. The fiber mats were first sterilized and then added to a 24-well plate and washed with PBS and medium before seeding the HMVECs, upon which H_2_O_2_ was added to the wells to reach a final concentration of 600 μM. The cells were incubated for another 16 h before performing live/dead staining to visualize viability. To quantify the ratio of live/dead cells, we first utilized NIS Viewer to apply an automatic look-up table (LUT) function to enhance the contrast of the images while preserving the image data (Figures 10 and S24).

Next, we used ImageJ to quantify the %live cells on each fiber mat (details can be found in the Experimental Section), and we determined that all assays on the electrospun fiber mats exhibited >80% live cells (Figure S25). These results are in contrast to those in Figure 7B, which indicate that cells without the presence of fiber mats exhibited ∼50% viability after the addition of 600 μM H_2_O_2_. We attribute the discrepancy of these results in 2D and 2.5D cell culture to the ability of integrins to mediate attachment and spreading, transducing signals that regulate cell growth, survival, and gene expression.^63^ Here in 2D cultures (Figure 7B), cells adhered flatly to the well plate with maximal H_2_O_2_ exposure and lacked ECM-mediated survival signals, which likely explains the increased cell death. In electrospun mats (Figure 10), the 2.5D structure likely activated integrin pathways, shielding the cells and reducing oxidative damage, a phenomenon that has been previously observed in 2.5D and 3D cell culture.^64^

**Figure 10:**
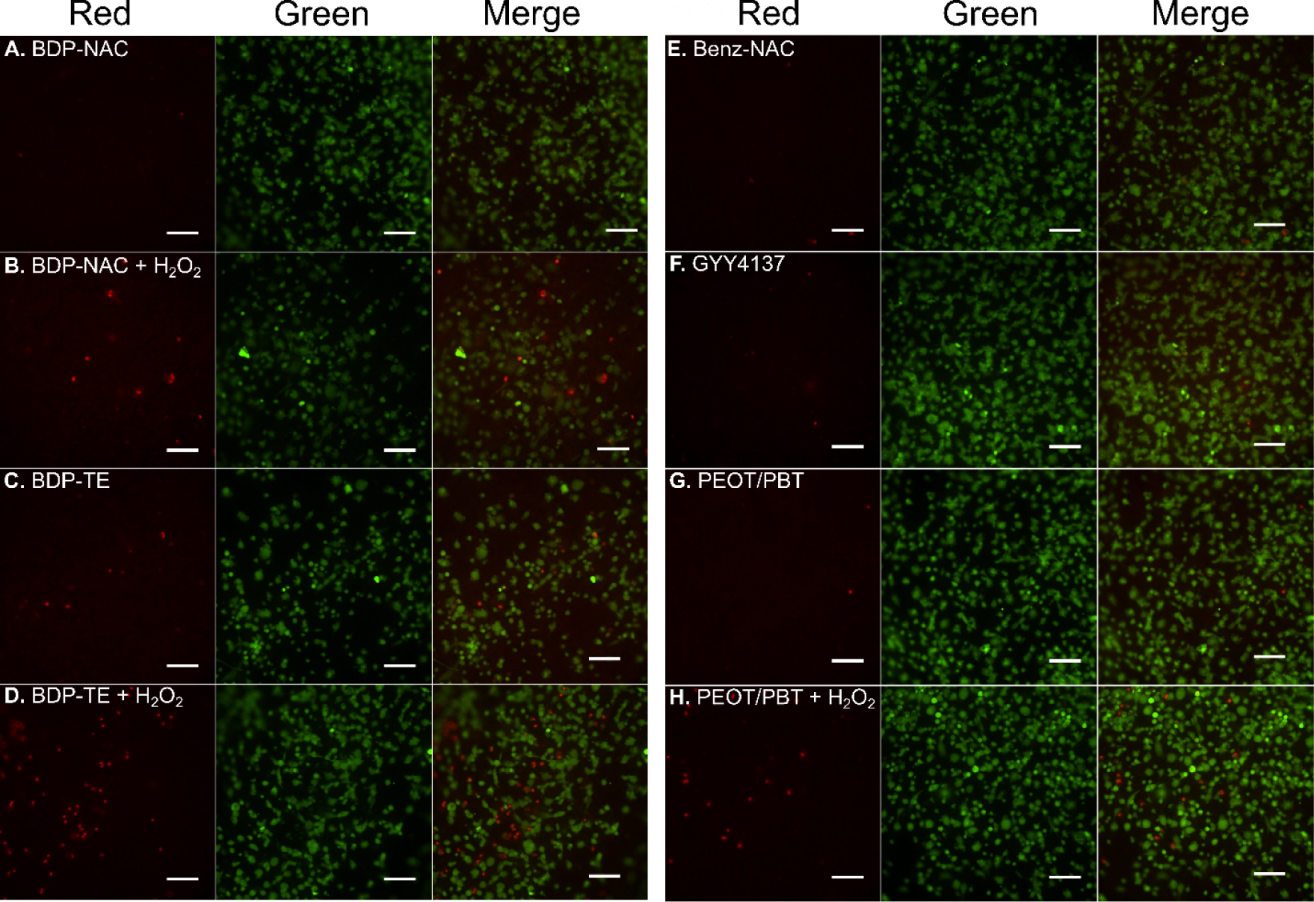
Live/dead staining of cell cultures on electrospun fiber mats containing prodrugs/controls, either with or without the addition of 600 μM H_2_O_2_. (A) BDP-NAC-doped PEOT/PBT, (B) BDP-NAC-doped PEOT/PBT + H_2_O_2_, (C) BDP-TE-doped PEOT/PBT, (D) BDP-TE -doped PEOT/PBT + H_2_O_2_, (E) Benz-NAC, (F) GYY4137, (G) PEOT/PBT, and (H) PEOT/PBT + H_2_O_2_. Scale bar = 100 μm. Calcein AM (green/live) and propidium iodide.

In summary, the addition of electrospun PEOT/PBT fiber mats increased cell viability by 30% after exposure to H_2_O_2_, and these results indicate that fiber mats, such as those reported here, have promise for rescuing endothelial cells from oxidative stress. However, it is difficult to make definitive comparisons among each group due to the large error bars in Figure S25. The variability among these measurements is a consequence of imaging the cells in a 2.5D matrix, which caused difficulties in segmenting and definitively counting individual cells in the red and green channels. Future studies of the RSSH donor system reported here will investigate tube formation on the fiber mats as well as *in ovo* studies to probe the ability of these mats to induce vasculogenesis. Additionally, future efforts include exploiting the versatility of the electrospinning process in order to further tune the release of RSSH donors, fiber mat porosity, fiber thickness, and fiber diameter in order to optimize the ability of this system to induce vasculogenesis.

## Conclusions

In summary, we developed RSSH-releasing polymer fiber mats using an electrospinning process. We found that all five PEOT/PBT fiber mat groups were hydrophilic and exhibited a random fiber morphology, and including RSSH/H_2_S donors in the fiber mat electrospinning procedure did not majorly affect physical properties. Small molecule release studies indicated that the fiber mats released 20–50% of the small molecules over 4 h. Each of the small molecules in this study was nontoxic in HMVECs up to 200 μM. In examining the small molecules alone in the presence of H_2_O_2_ to mimic oxidative stress, **BDP-NAC** partially restored tube formation capability and outperformed the H_2_S donor Na_2_S. Lastly, we found that HMVECs exhibited high viability on the electrospun scaffolds, even when exposed to high levels of H_2_O_2_ (600 μM). These early experiments suggest that physically incorporated **BDP-NAC** could also rescue cells from oxidative stress on fiber mats. This study shows the potential of this approach to facilitate vascularization. Future studies will leverage electrospinning to further study vasculogenesis via *in vitro* and *ex vivo* assays in order to probe the effects of systems such as these on the ability of vessels to spread into injured sites. Finally, this work highlights alternatives to VEGF, with H_2_S and RSSH donors as promising candidates. With the combination of increased survival under oxidative stress and the ability to maintain vascularization in an early tube-forming assay, the small molecule-releasing fiber mats could have a promising path forward for tissue engineering approaches that need vasculogenesis under oxidative conditions.

## Supplementary Material



## Notes

Additional experimental procedures; SEM micrographs; contact angle images and measurements; FTIR analysis; film thickness measurements; mechanical analysis; small molecule release kinetics from electrospun fiber mats; cell culture experimental procedures and images (PDF)

## Abbreviations

RSSH: persulfides
H_2_S: hydrogen sulfide
HMVECs: human microvascular endothelial cells
H_2_O_2_: hydrogen peroxide
ROS: reactive oxygen species
VEGF: vascular endothelial growth factor
PCL: poly­(caprolactone)
HUVEC: human umbilical vein endothelial cell
PEOT/PBT: poly­(ethylene oxide terephthalate)/poly­(butylene terephthalate)
HFIP: hexafluoroisopropanol
SEM: scanning electron microscopy
UV–vis: ultraviolet visible spectroscopy
D_2_O: deuterium oxide
D_3_PO_4_: phosphoric acid-*d*_3_
DMSO: dimethyl sulfoxide
Na_2_S: sodium sulfide
PBS: phosphate buffered saline
ECM: extracellular matrix
DSC: differential scanning calorimetry
