# Supplementary material for: Electrospun Polymer Fiber Mats for Persulfide Prodrug Delivery
Source: Biomacromolecules. 2025 Aug 1;26(9):6057–69. doi: 10.1021/acs.biomac.5c00975 (PMC12421669; doi:10.1021/acs.biomac.5c00975)
Supplement: Supplementary file 1 [file bm5c00975_si_001.pdf]

Electrospun polymer fiber mats for persulfide prodrug delivery

Sarah N. Swilley[1,2], Hao Wu[1], Clarissa Tomasina[1], Lorenzo Moroni[1], Paul Wieringa[1*], and Matthew B. Baker,[1,3*] John B. Matson[2*]

[1]MERLN Institute for Technology-inspired Regenerative Medicine, Complex Tissue Regeneration Department, Maastricht University, P.O. Box 616, 6200MD, Maastricht, The Netherlands

[2]Department of Chemistry, Virginia Tech Center for Small molecule Discovery, Macromolecules Innovation Institute, 1040 Drillfield Dr, Blacksburg, VA 24061, USA

[3]MERLN Institute for Technology-inspired Regenerative Medicine, Instructive Biomaterials Engineering Department, Maastricht University, P.O. Box 616, 6200MD, Maastricht, The Netherlands

*Corresponding authors: p.wieringa@maastrichtuniversity.nl, m.baker@maastrichtuniversity.nl, jbmatson@vt.edu

Supporting Information

Table of Contents:

Materials and Methods
Electrospun Fiber Mat Fabrication 3
Scanning electron microscopy (SEM) 4
Contact Angle and DSC Analysis 10
FTIR analysis 11
Mechanical Analysis 12
Release of Small Molecules from Electrospun Fiber Mats 14
Cell Culture 15
- **Live/dead Staining** 15
- **PrestoBlue and DNA Assay** 17
- **Live/dead staining after 16 h exposure to varying concentrations of H_2O_2** 18
- **Live/dead staining after 16 h exposure to 600 or 800 µM H_2O_2 and/or 200 µM of Na_2S, or BDP-NAC** 19
- **Angiogenesis assay after 16 h exposure to 600 µM H_2O_2 and/or 200 µM of Na_2S, or BDP-NAC both with and without VEGF present in the growth medium** 21
- **Cell Culture on Electrospun Fiber mats and Quantification of Cell Viability on Fiber Mats** 23

Materials and Methods

All starting materials were used as received from commercial vendors unless otherwise specified. Yields refer to compounds as isolated after purification unless otherwise stated. Thin-layer chromatography (TLC) was performed on aluminum-backed silica plates and visualized by UV or iodine. ^{1}H NMR spectroscopy was performed on a 700 or 500 MHz Bruker, or an Agilent 400 MHz spectrometer at rt. ^{1}H NMR chemical shifts are reported in ppm relative to internal solvent resonances. Statistical analysis was calculated via one-way ANOVA followed by Tukey post-hoc tests.

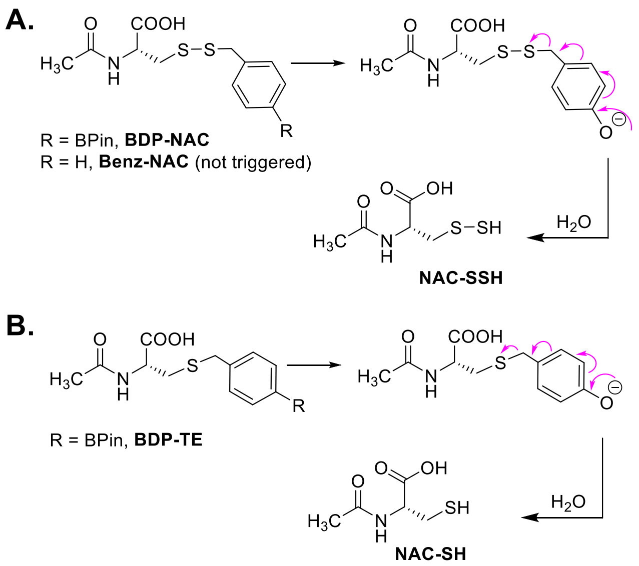

Scheme S1: Abbreviated mechanism showing release of A) *N*-Acetylcysteine (NAC) RSSH derivative (**NAC-SSH**) from disulfide-based donors, and B) Release of **NAC-SH** from thioether-based donors.

Electrospun Fiber Mat Fabrication

Fiber mats were composed of PEOT/PBT, commercially available as Polyactive™ **Fig. 2A**, 300 PEOT/PBT 55:45 where 300 refers to the initial M_w of PEG used in the copolymer reaction and 55:45 to the weight ratio between PEOT and PBT after copolymerization.

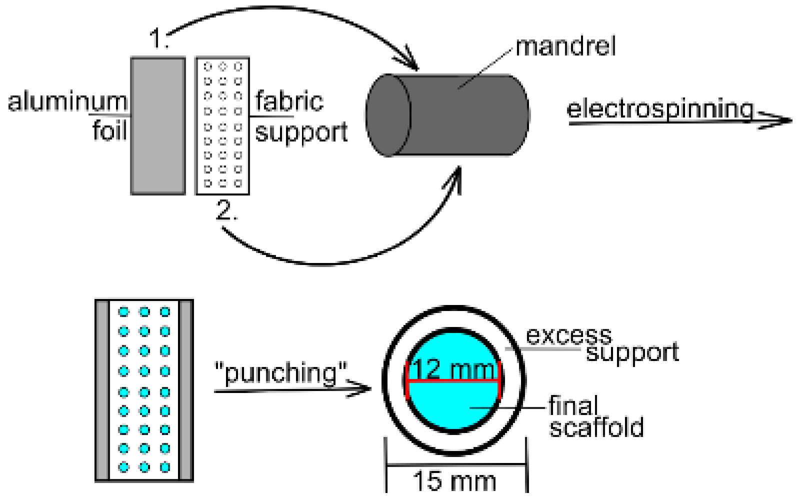

Fig. S1. Cartoon depicting the electrospinning and punching process to fabricate the electrospun fiber mats.

Scanning electron microscopy (SEM)

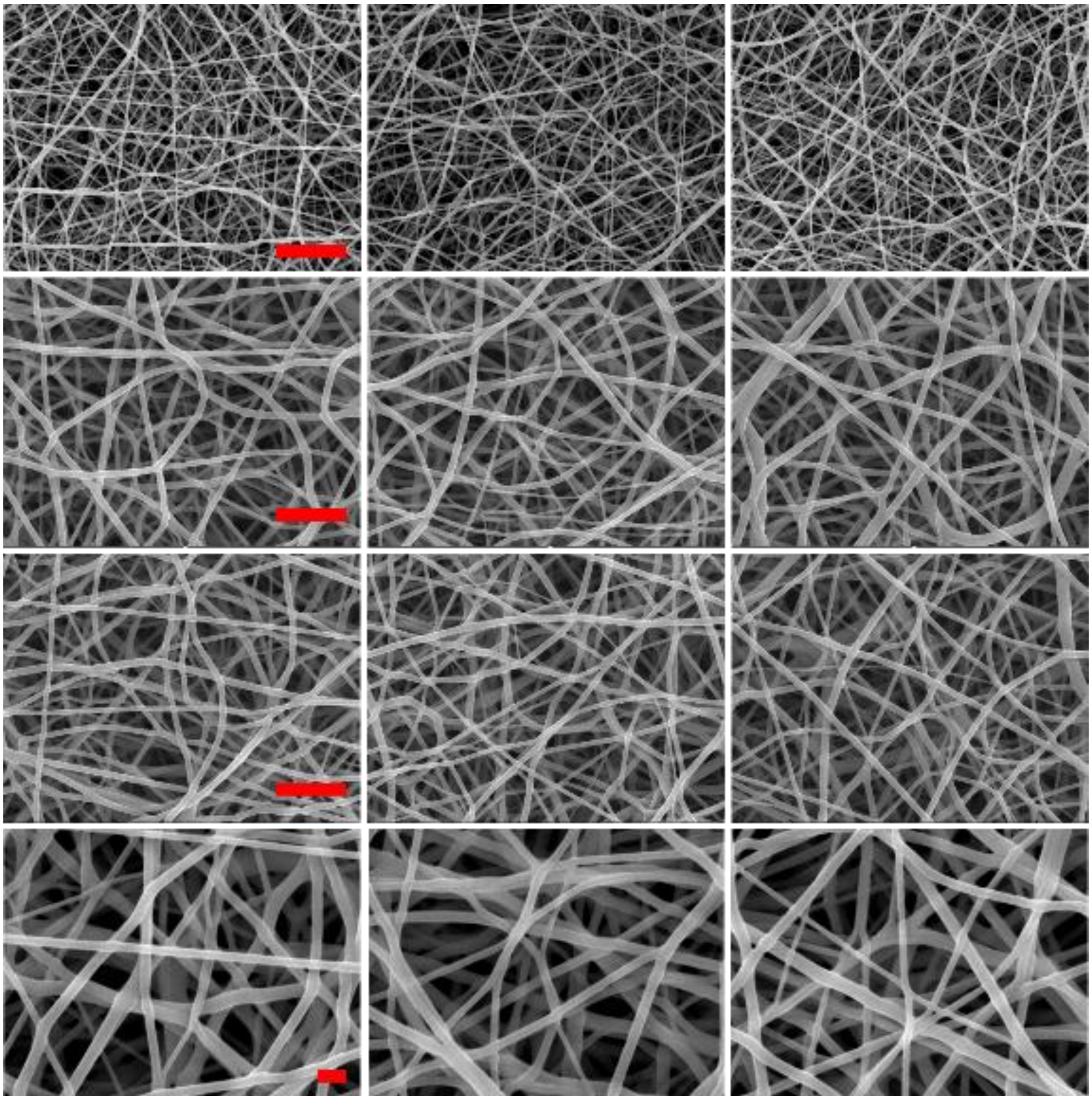

Fig. S2: SEM micrographs of **BDP-NAC** doped PEOT/PBT at 2,500X magnification (top row, scalebar is 10 μm), at 5,000X magnification (second and third rows, scalebar is 5 μm), and at 10,000X magnification (bottom row, scalebar is 1 μm).

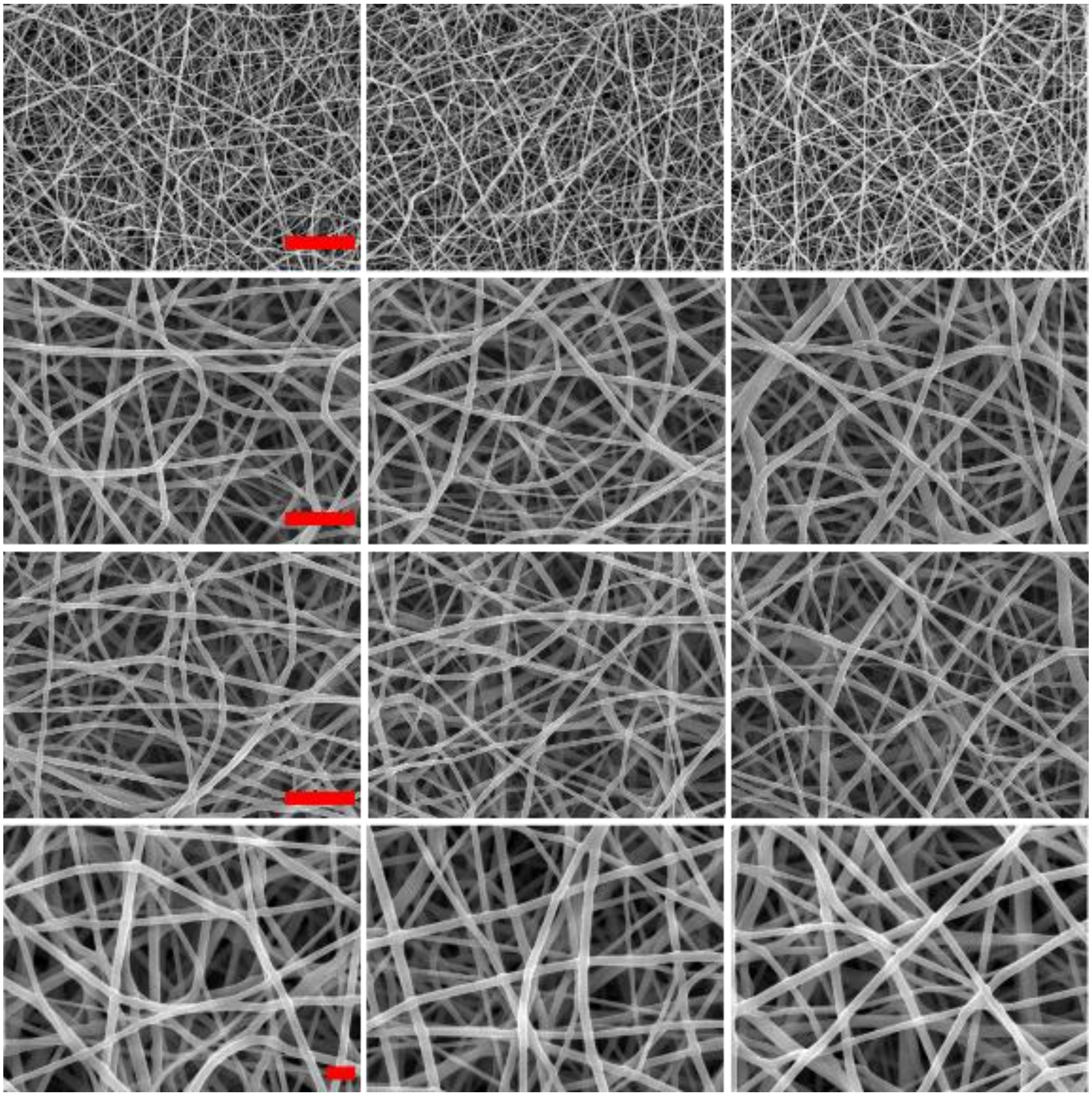

Fig. S3: SEM micrographs of **BDP-TE** doped PEOT/PBT at 2,500X magnification (top row, scalebar is 10 μm), at 5,000X magnification (second and third rows, scalebar is 5 μm), and at 10,000X magnification (bottom row, scalebar is 1 μm).

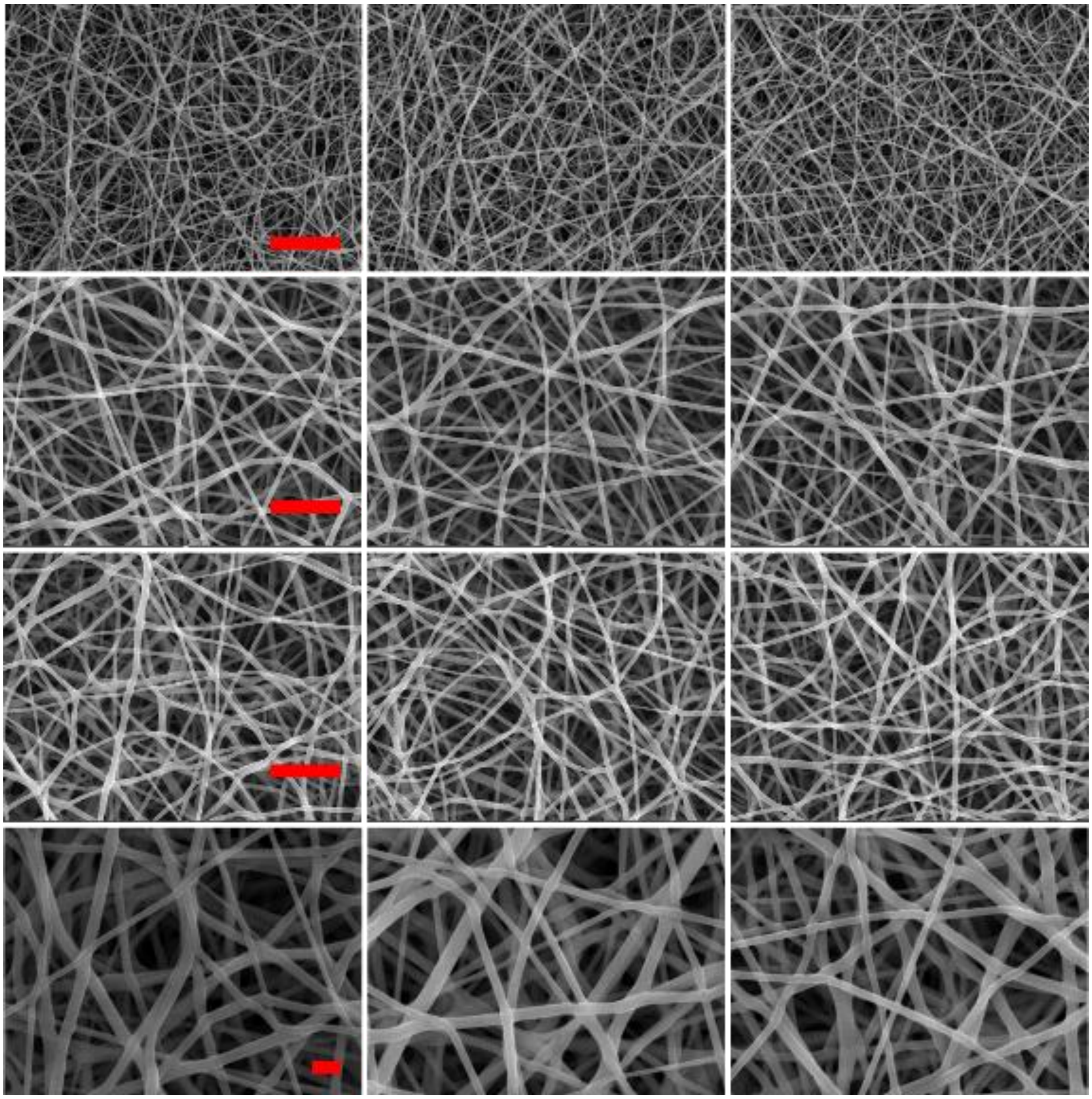

Fig. S4: SEM micrographs of **Benz-NAC** doped PEOT/PBT at 2,500X magnification (top row, scalebar is 10 µm), at 5,000X magnification (second and third rows, scalebar is 5 µm), and at 10,000X magnification (bottom row, scalebar is 1 µm).

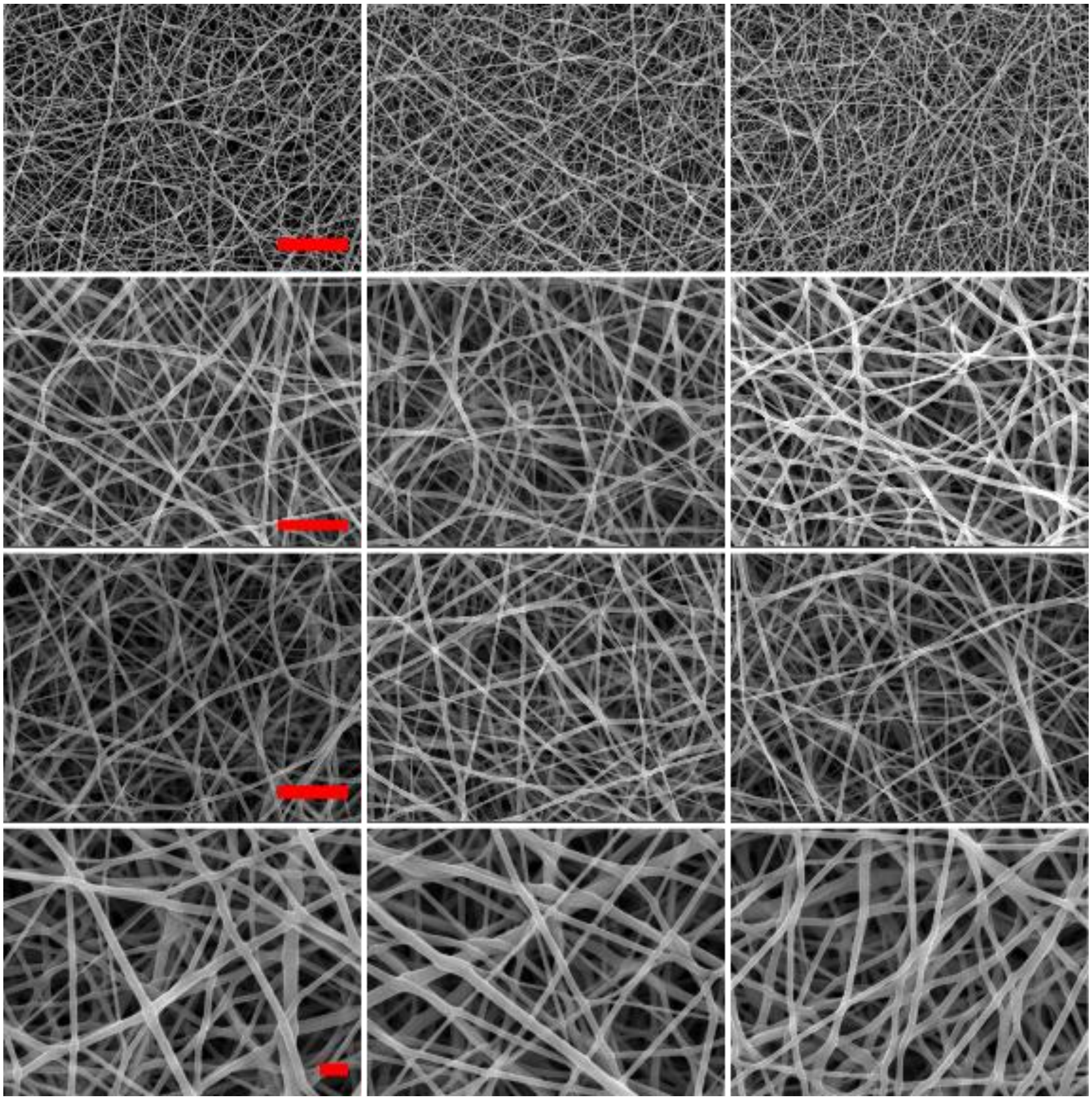

Fig. S5: SEM micrographs of **GYY4137** doped PEOT/PBT at 2,500X magnification (top row, scalebar is 10 µm), at 5,000X magnification (second and third rows, scalebar is 5 µm), and at 10,000X magnification (bottom row, scalebar is 1 µm).

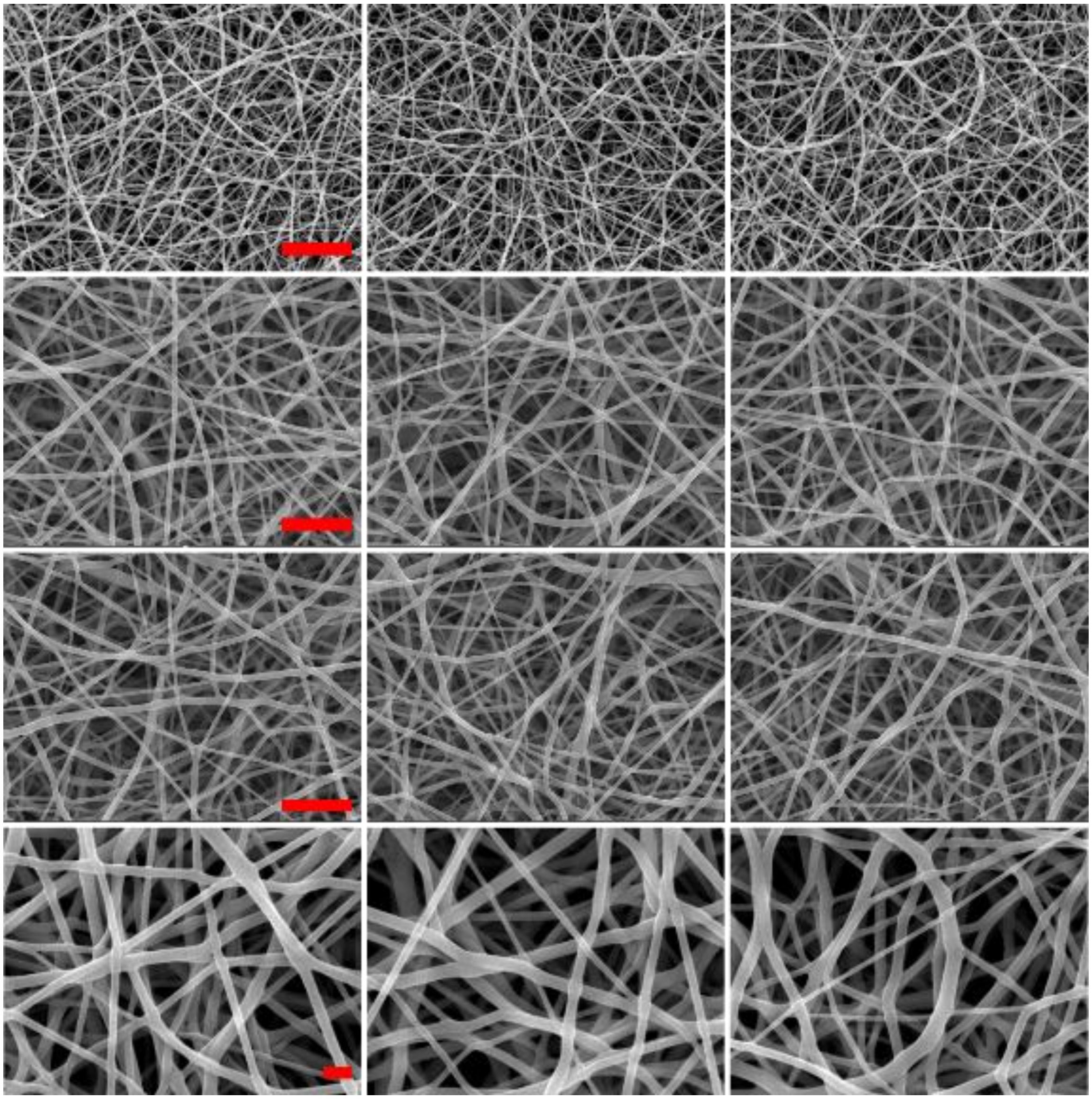

Fig. S6: SEM micrographs of PEOT/PBT at 2,500X magnification (top row, scalebar is 10 µm), at 5,000X magnification (second and third rows, scalebar is 5 µm), and at 10,000X magnification (bottom row, scalebar is 1 µm).

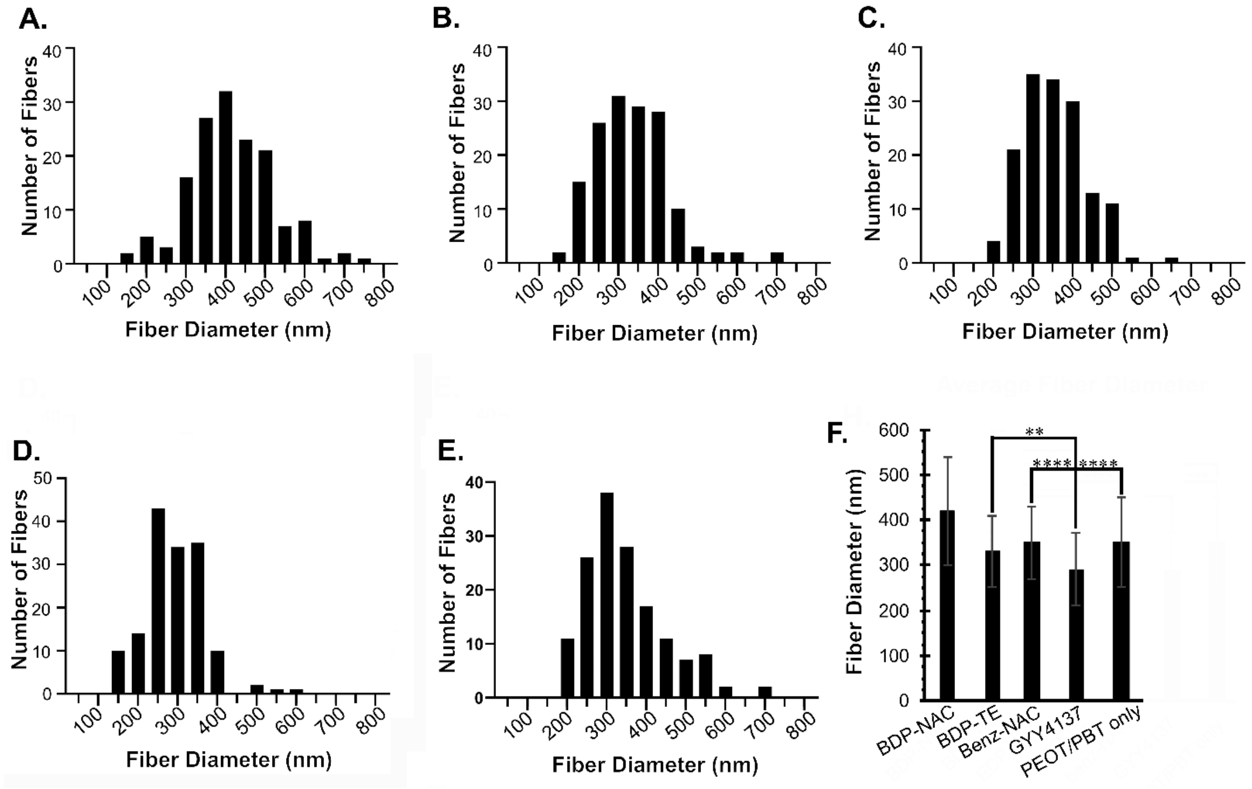

Fig. S7: Histograms (A-E) of fiber diameters for each type of fiber mat and bar graph (H) depicting fiber diameters between different groups, n = 150 for all analyses (A) **BDP-NAC** doped PEOT/PBT, (B) **BDP-NAC** doped PEOT/PBT, (C) **Benz-NAC** doped PEOT/PBT, (D) **GYY4137** doped PEOT/PBT, (E) PEOT/PBT (F) bar graph depicting fiber diameters of the five types of fiber mat with statistics. Statistical analysis was determined via one-way ANOVA followed by Tukey post-hoc tests. **** indicates $p < 0.0001$, *** indicates $p < 0.001$, ** indicates $p < 0.01$. * indicates $p < 0.05$. **BDP-NAC** *films were found to have a significance of* $p < 0.0001$ *between all groups*. **** indicates $p < 0.0001$, *** indicates $p < 0.001$, ** indicates $p < 0.01$. * indicates $p < 0.05$.

Contact Angle and DSC Analysis

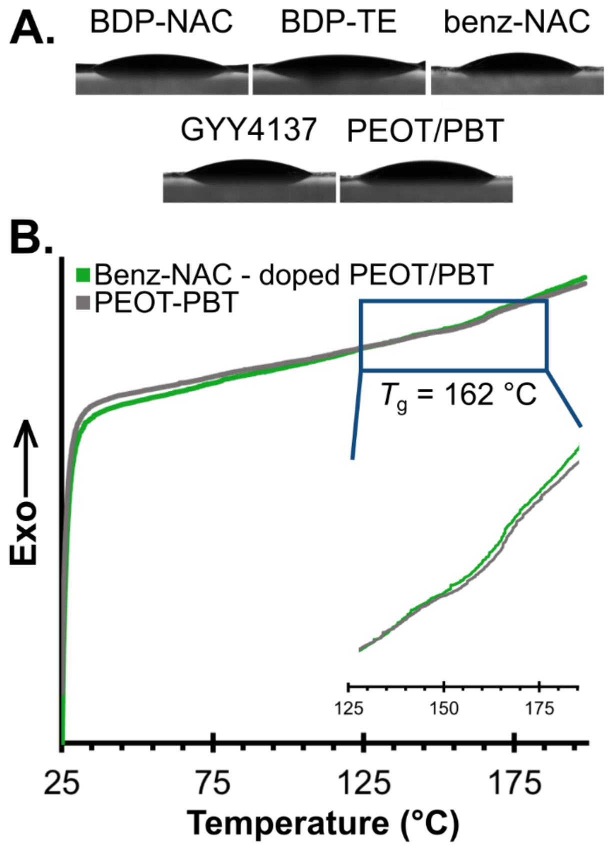

Fig. S8: A) Still-shot images from contact angle analysis. Each image shown is the first frame from the corresponding video in which the water droplet makes contact with the fiber mat's surface. The θ left values for each fiber mat were calculated via ImageJ. **BDP-NAC-**doped PEOT/PBT 148°; **BDP-NAC-**doped PEOT/PBT 158.7°, **Benz-NAC-**doped PEOT/PBT 146.4°, **GYY4137-**doped PEOT/PBT 151.8°, and PEOT/PBT 148.7°. B) DSC thermograms of **Benz-NAC**-doped PEOT-PBT and pristine PEOT-PBT; both samples were found to have a T_m of 162 °C.

FTIR analysis

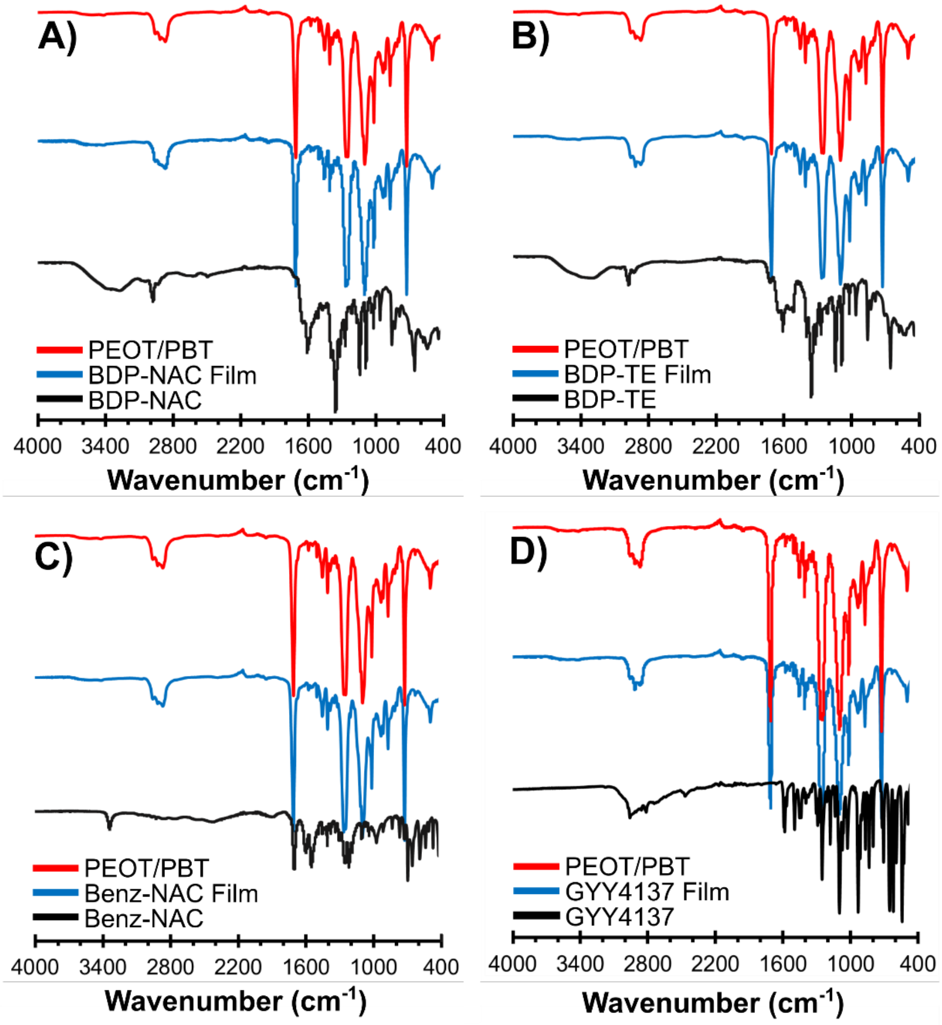

Figure S9: FTIR spectra of the small molecules (black traces) that were electrospun into the PEOT/PBT fiber mats as compared to the spectra of the doped fiber mats (blue traces) as well as PEOT/PBT (red traces). A) **BDP-NAC** samples, B) **BDP-TE** samples, C) **Benz-NAC** samples, and D) **GYY4137** samples.

Due to the small amount of RSSH donors that were incorporated into the polymer fiber mats we were unable to identify any characteristic peaks of the RSSH donors when examining the spectra of the small molecules and the doped fiber mats.

Mechanical Analysis

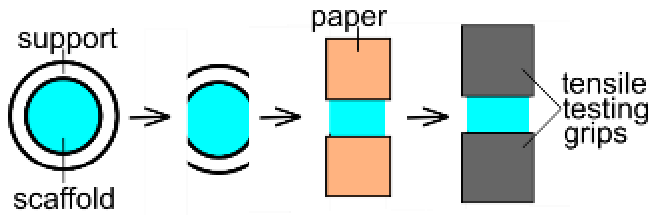

Fig. S10: Cartoon depicting the sample preparation of electrospun fiber mats for tensile testing.

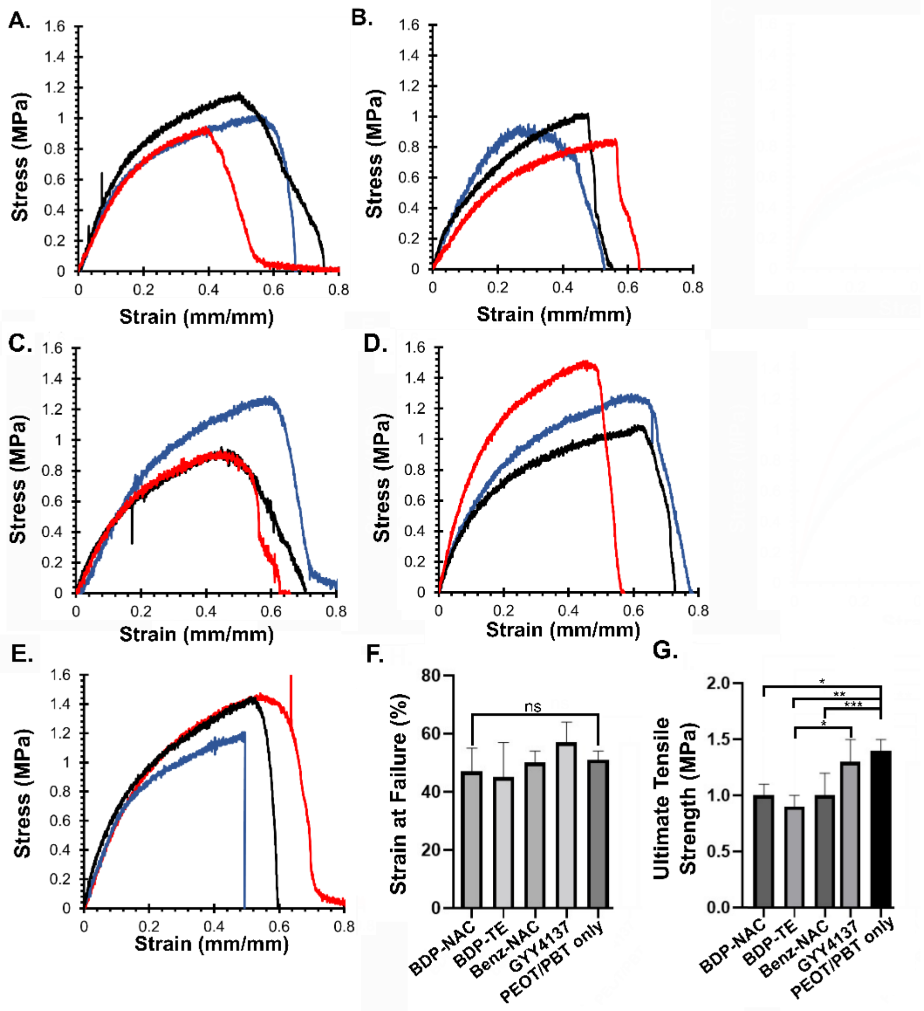

Fig. S11: Stress-strain curves for each electrospun fiber mat. A) **BDP-NAC** doped PEOT/PBT, B) **BDP-TE** doped PEOT/PBT, C) **Benz-NAC** doped PEOT/PBT, D) **GYY4137** doped PEOT/PBT, E) PEOT/PBT, F) statistical analysis of strain at failure among all groups, no significance was determined, and G) statistical analysis of ultimate tensile strength among all groups tested. Statistical analysis was determined via one-way ANOVA followed by Tukey post-hoc tests. *** indicates $p < 0.001$, ** indicates $p < 0.01$. * indicates $p < 0.05$. n = 3

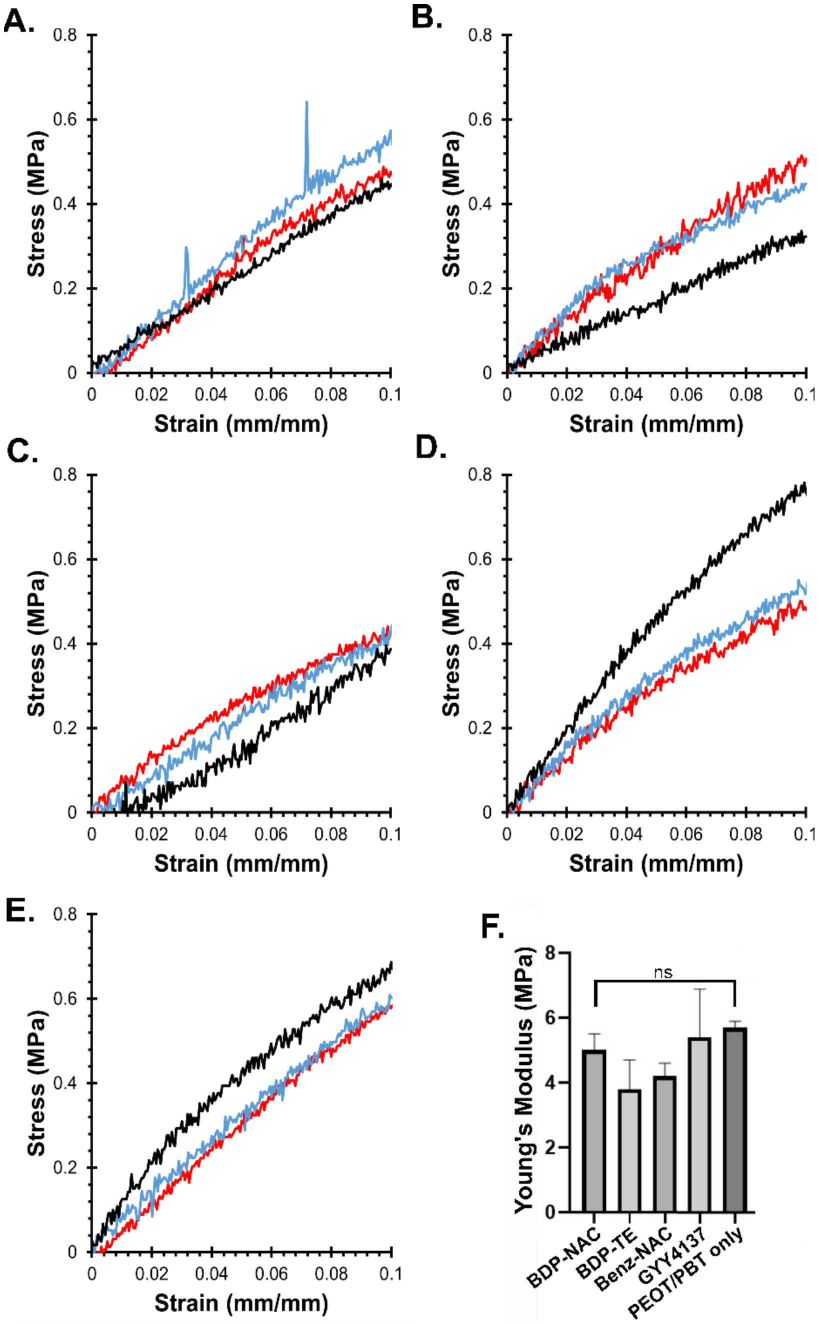

Fig. S12: Zoomed in portion from **Fig. S13** highlighting the linear region of each curve. A) **BDP-NAC** doped PEOT/PBT, B) **BDP-TE** doped PEOT/PBT, C) **Benz-NAC** doped PEOT/PBT, D) **GYY4137** doped PEOT/PBT, E) PEOT/PBT, and F) statistical analysis among all groups tested with no significance found. Statistical analysis was determined via one-way ANOVA followed by Tukey post-hoc tests. n = 3

Release of Small Molecules from Electrospun Fiber Mats

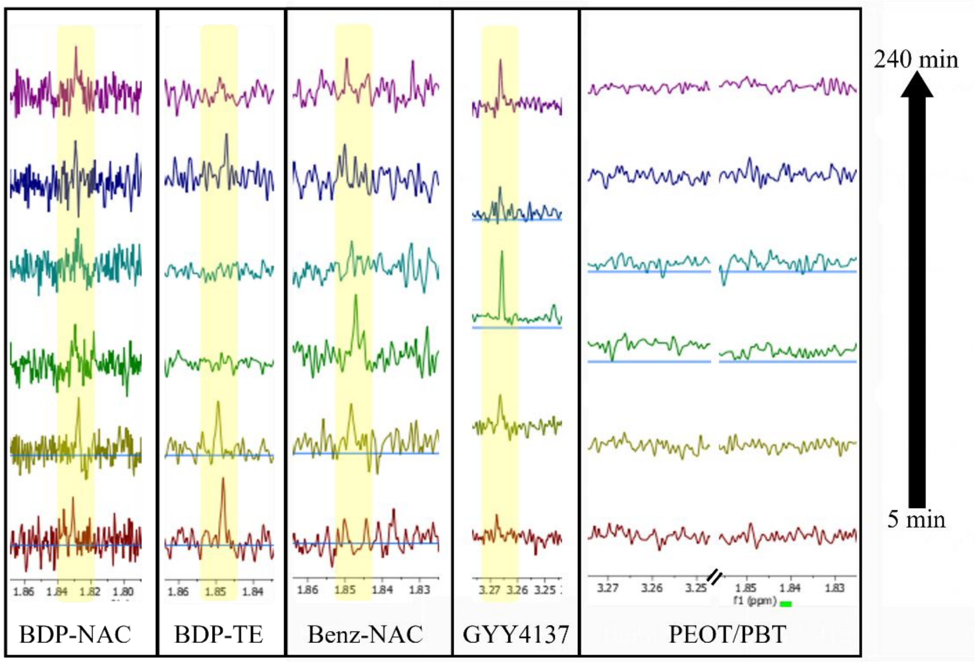

Fig. S13: NMR spectra from which the drug release kinetics were calculated. The peaks highlighted in yellow were integrated with respect to the maleic acid internal standard.

Cell Culture

Live/dead Staining

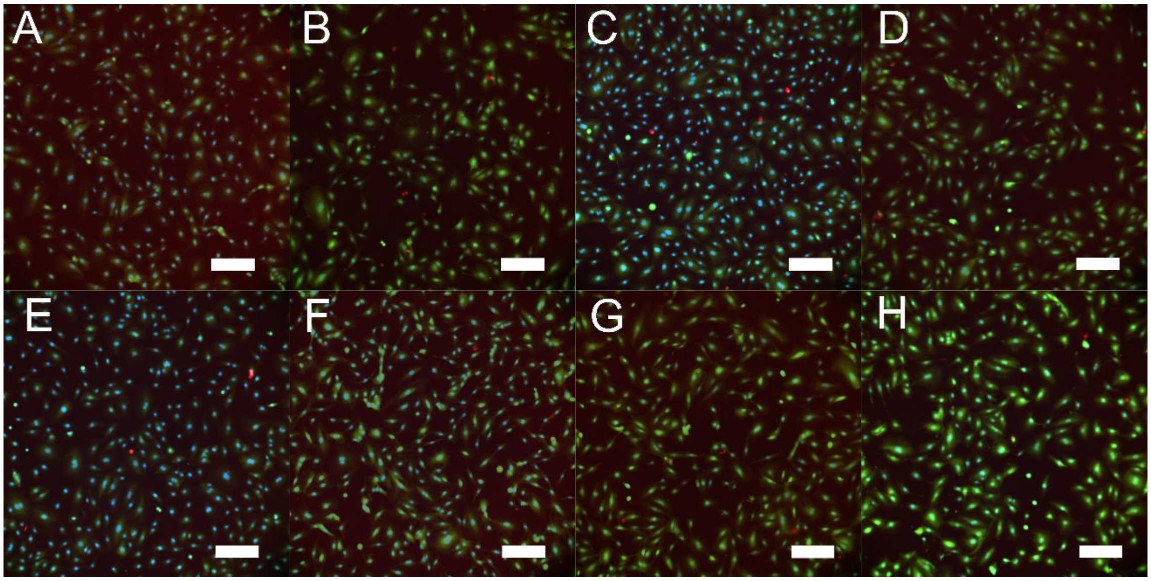

Fig. S14: Representative live/dead images of HMVECs after exposure to various compounds and 100 μM H_2O_2 after 1 d. A). control, B). 100 μM H_2O_2, C). 1% DMSO in media, D). 200 μM Na_2S, E). 200 μM **GYY4137** , F). 200 μM **BDP-NAC**, G). 200 μM **BDP-TE**, and H). 200 μM **Benz-NAC**. Panels D-H also had 1% DMSO in media in order to solubilize the small molecules. Scale bar is 200 μm. Calcein AM (green/live), propidium iodide (red/dead), and Hoechst (blue/nucleus).

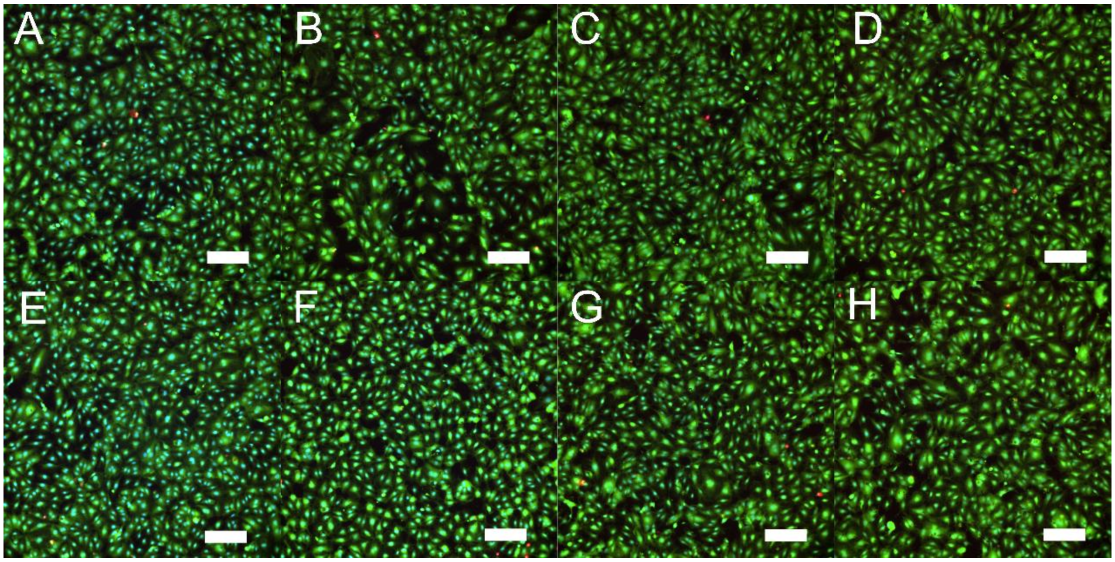

Fig. S15: Live/dead images of HMVECs after exposure to various compounds and 100 µM H_2O_2 after 3 d. A). control, B). 100 µM H_2O_2, C). 1% DMSO in media, D). 200 µM Na_2S, E). 200 µM **GYY4137** , F). 200 µM **BDP-NAC**, G). 200 µM **BDP-TE**, and H). 200 µM **Benz-NAC**. Panels D-H also had 1% DMSO in media in order to solubilize the small molecules. Scale bar is 200 µm. Calcein AM (green/live), propidium iodide (red/dead), and Hoechst (blue/nucleus).

PrestoBlue and DNA Assay

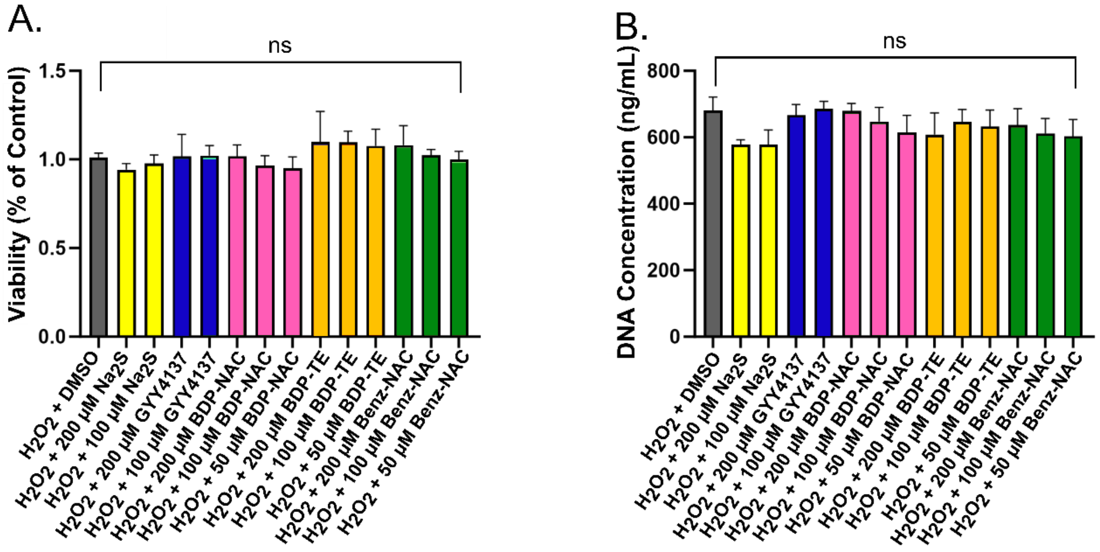

Fig. S16: Presto blue viability assay of HMVECs (left) after exposure to different compounds at varying concentrations along with 100 µM H_2O_2 after 1 d. DMSO was diluted to 1% in cell media before use. Viability was normalized to the H_2O_2 group. Concentration of DNA present in HMVECs (right) after 3 d. DNA concentration was determined via an Invitrogen, C7026 DNA assay kit. Statistical analysis was determined via one-way ANOVA followed by Tukey post-hoc tests. Error bars represent the standard deviation of the mean for 3 independent experiments with n = 3 for each experiment.

Live/dead staining after 16 h exposure to varying concentrations of H_2O_2

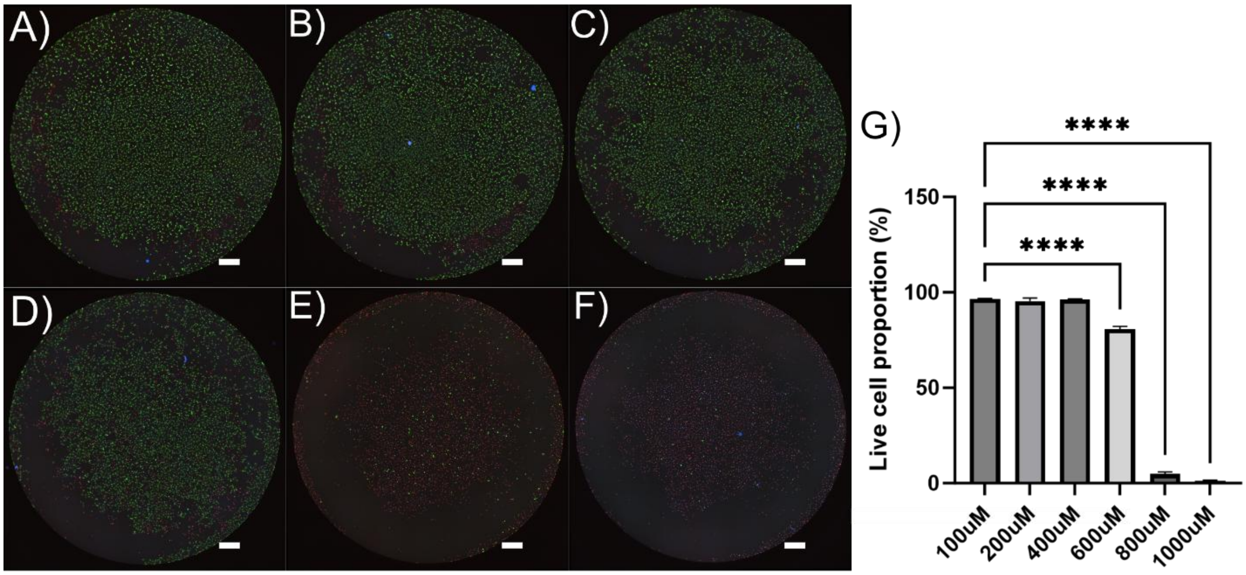

Fig. S17: HMVECs treated with various concentrations of H_2O_2. A) 100 µM H_2O_2, B) 200 µM H_2O_2, C) 400 µM H_2O_2, D) 600 µM H_2O_2, E) 800 µM H_2O_2, F) 1000 µM H_2O_2. Scale bar is 500 µm. Calcein AM (green/live), propidium iodide (red/dead), and Hoechst (blue/nucleus). G) Bar graph quantifying the live cell proportion as compared to untreated cells. Statistical analysis was determined via one-way ANOVA followed by Tukey post-hoc tests. **** indicates $p < 0.0001$, *** indicates $p < 0.001$, ** indicates $p < 0.01$. * indicates $p < 0.05$. Error bars represent the standard deviation of the mean for 3 independent experiments with n = 3 for each experiment.

Live/dead staining after 16 h exposure to 600 or 800 µM H_2O_2 and/or 200 µM of Na_2S, or BDP-NAC

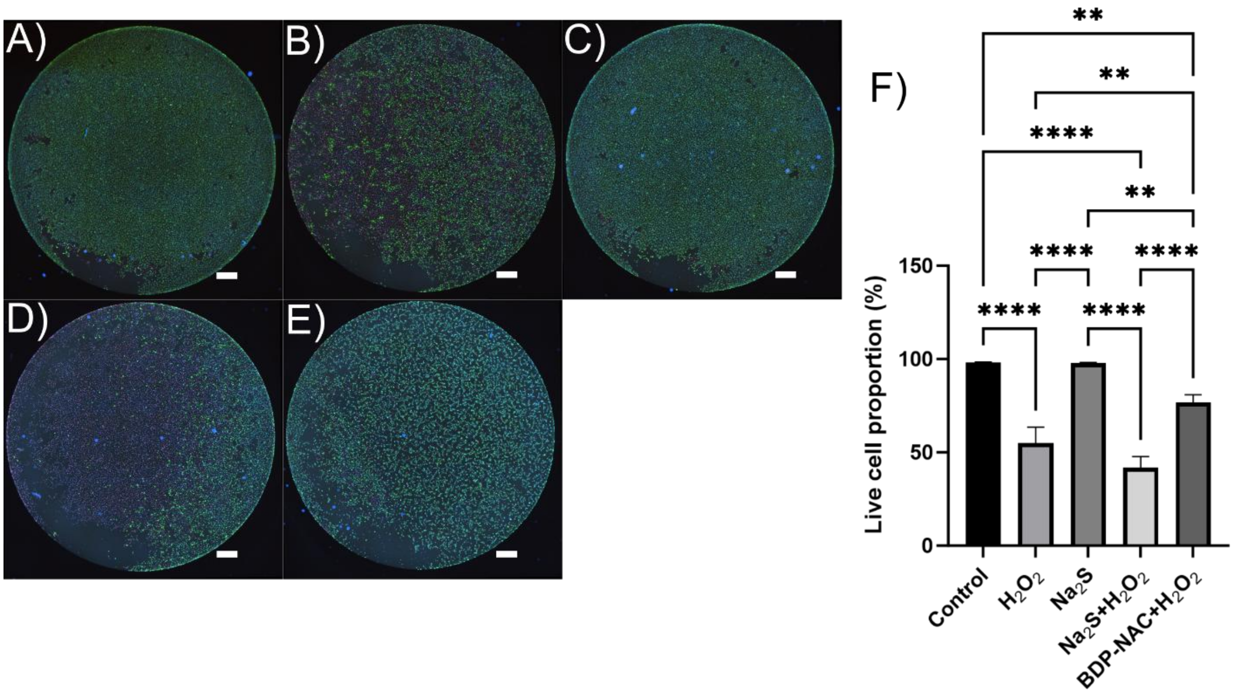

Fig. S18: Live/dead staining of HMVECs after exposure to different small molecules. A) Control, B) 600 µM H_2O_2 only, C) 200 µM Na_2S, D) 200 µM Na_2S + 600 µM H_2O_2, and E) 200 µM **BDP-NAC** + 600 µM H_2O_2. Scale bar is 500 µm. Calcein AM (green/live), propidium iodide (red/dead), and Hoechst (blue/nucleus). F) Bar graph quantifying the live cell proportion as compared to untreated cells. Statistical analysis was determined via one-way ANOVA followed by Tukey post-hoc tests. **** indicates $p < 0.0001$, *** indicates $p < 0.001$, ** indicates $p < 0.01$. * indicates $p < 0.05$. Error bars represent the standard deviation of the mean for 3 independent experiments with n = 3 for each experiment.

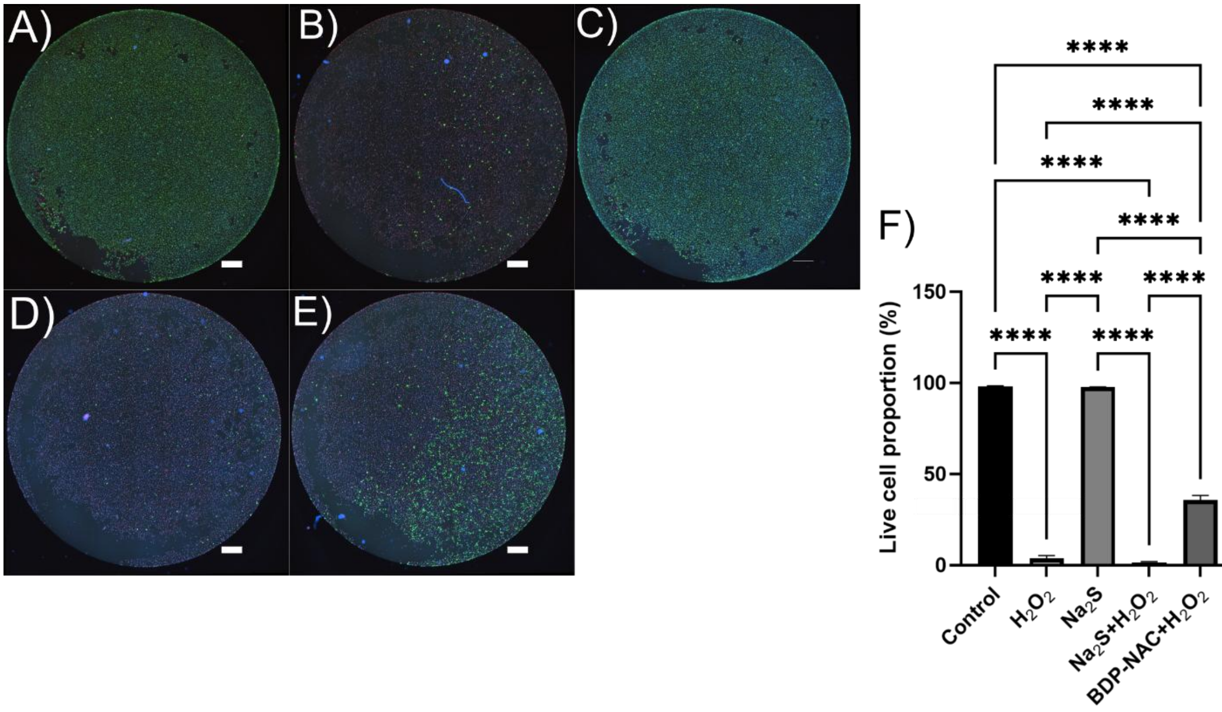

Fig. S19: Live/dead staining of HMVECs after exposure to 800 μM H_2O_2 (B-F) with added small mlecules. A) Control, B) 800 μM H_2O_2, C) 200 μM Na_2S, D) 200 μM Na_2S + 800 μM H_2O_2 , and E) 200 μM **BDP-NAC** + 800 μM H_2O_2. Scale bar is 500 μm. Calcein AM (green/live), propidium iodide (red/dead), and Hoechst (blue/nucleus). G) Bar graph quantifying the live cell proportion as compared to untreated cells. Statistical analysis was determined via one-way ANOVA followed by Tukey post-hoc tests. **** indicates $p < 0.0001$, *** indicates $p < 0.001$, ** indicates $p < 0.01$. * indicates $p < 0.05$. Error bars represent the standard deviation of the mean for 3 independent experiments with n = 3 for each experiment.

Angiogenesis assay after 16 h exposure to 600 μM H_2O_2 and/or 200 μM of Na_2S, or BDP-NAC both with and without VEGF present in the growth medium

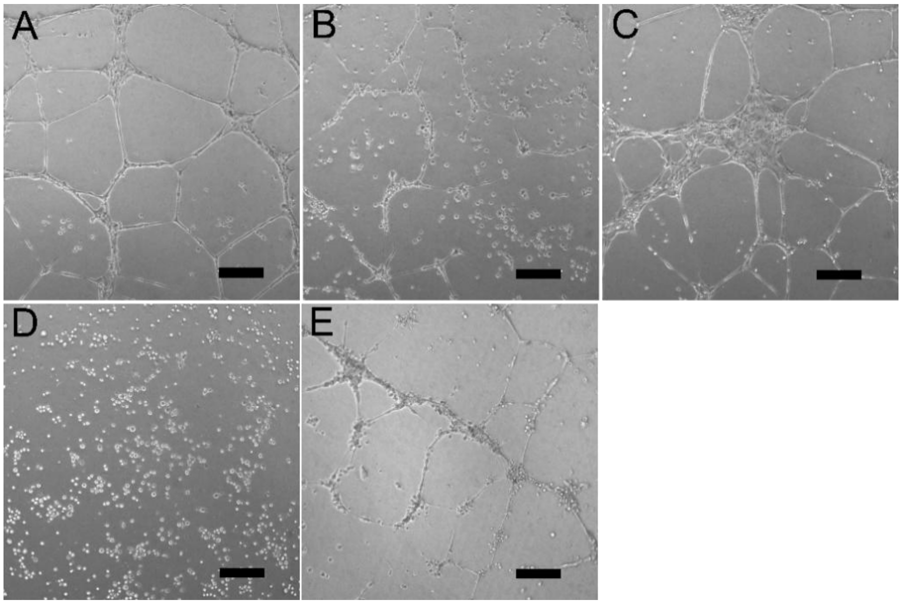

Fig. S20: Brightfield images of HMVECs after seeding on Geltrex, utilizing 600 μM H_2O_2 and 200 μM of each small molecule tested in media without VEGF. A). Control, B). 600 μM H_2O_2, C). 200 μM Na_2S, D). 200 μM Na_2S + 600 μM H_2O_2, and E). 200 μM **BDP-NAC** + 600 μM H_2O_2. Scale bar is 200 μm.

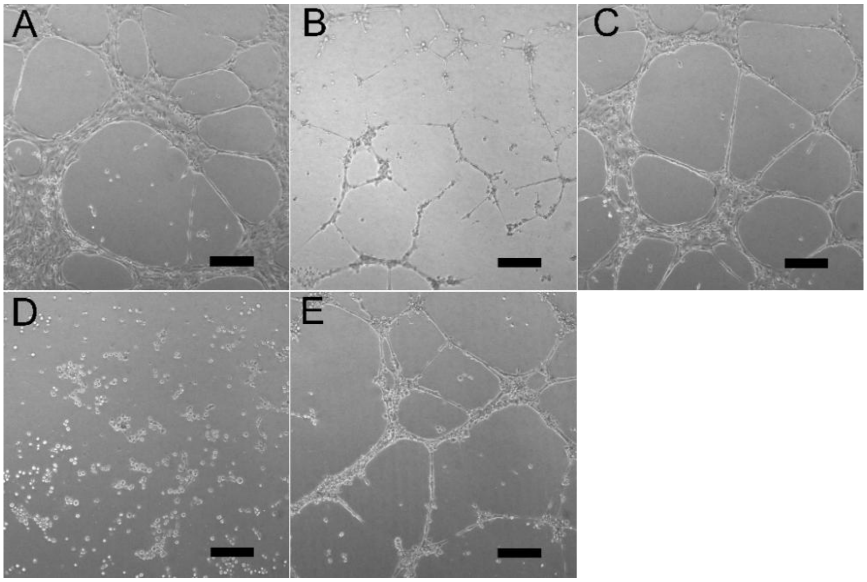

Fig. S21: Brightfield images of HMVECs after seeding on Geltrex, utilizing 600 μM H_2O_2 and 200 μM of each small molecule tested in media with VEGF. A). Control, B). 600 μM H_2O_2, C). 200 μM Na_2S, D). 200 μM Na_2S + 600 μM H_2O_2, and E). 200 μM **BDP-NAC** + 600 μM H_2O_2. Scale bar is 200 μm.

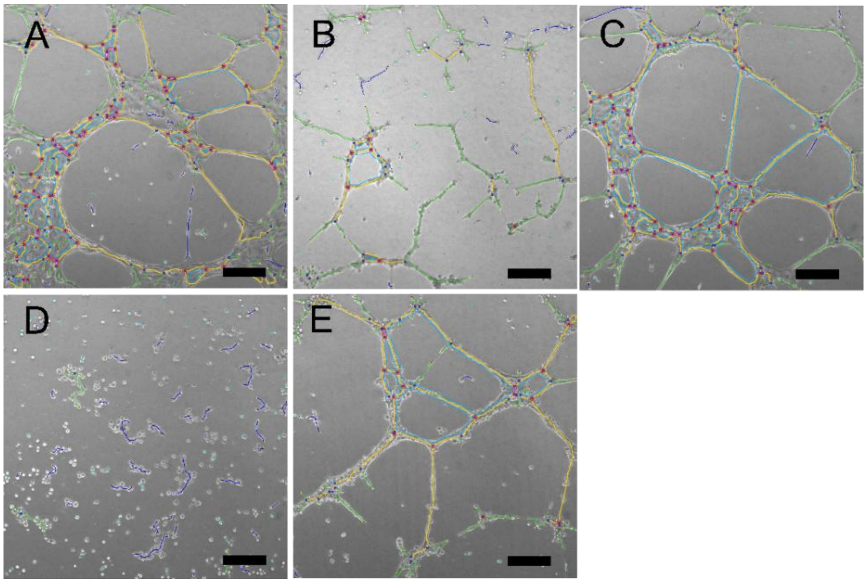

Fig. S22: Analyzed angiogenesis images using Fiji software; 600 µM H_2O_2 and 200 µM of each small molecule tested in media with VEGF. A). Control, B). 600 µM H_2O_2, C). 200 µM Na_2S, D). 200 µM Na_2S + 600 µM H_2O_2, and E). 200 µM **BDP-NAC** + 600 µM H_2O_2. Scale bar is 200 µm. Extremities: red dot; junctions: blue circle; branches: green line; segments: yellow line, meshes: blue area.

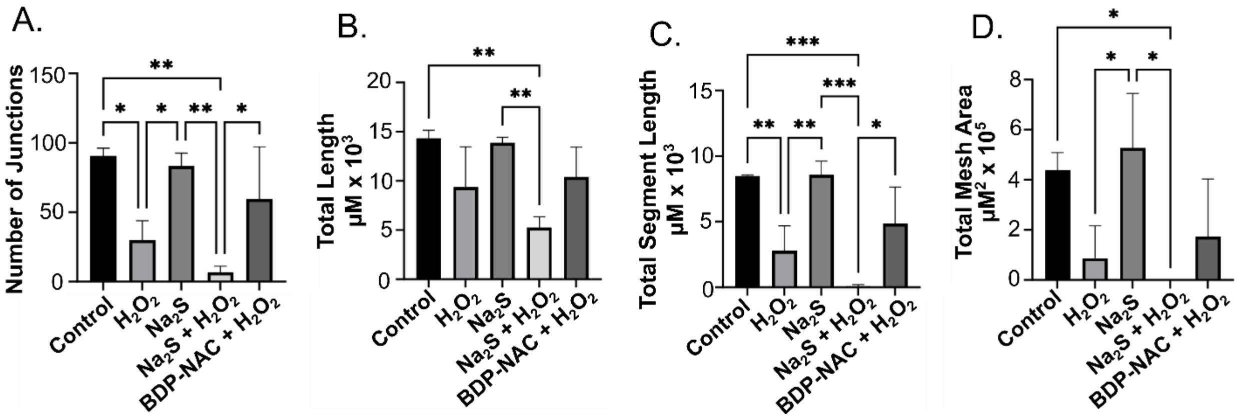

Fig. S23: Bar graphs used to quantify the A). number of junctions, B). total length, C). total segment length, and D). total mesh area of HMVECs utilizing 600 µM H_2O_2 and 200 µM of each small molecule tested in media with VEGF. Statistical analysis was determined via one-way ANOVA followed by Tukey post-hoc tests. **** indicates $p < 0.0001$, *** indicates $p < 0.001$, ** indicates $p < 0.01$. * indicates $p < 0.05$. Error bars represent the standard deviation of the mean for 3 independent experiments with n = 3 for each experiment.

Cell Culture on Electrospun Fiber mats and Quantification of Cell Viability on Fiber Mats

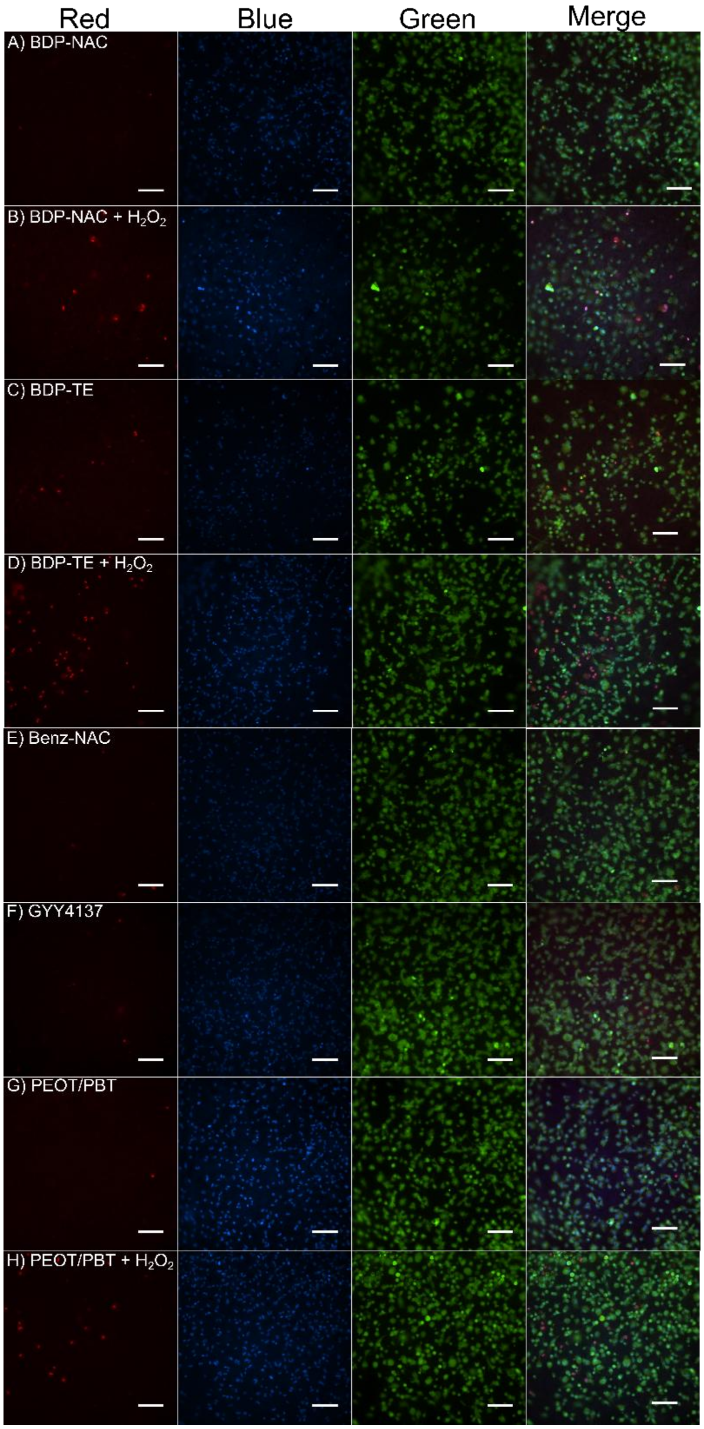

Figure S24: Live/dead staining of cells cultures on electrospun fiber mats containing prodrugs/controls either with or without the addition of 600 μM H_2O_2. A) **BDP-NAC**-doped

PEOT/PBT, B) **BDP-NAC-**doped PEOT/PBT + H_2O_2, C) **BDP-TE**-doped PEOT/PBT, D) **BDP-TE -**doped PEOT/PBT + H_2O_2 E) **Benz-NAC**, F) **GYY4137** G) PEOT/PBT, and H) PEOT/PBT + H_2O_2. Scalebar = 100 µm. Calcein AM (green/live), propidium iodide (red/dead), and Hoechst (blue/nucleus).

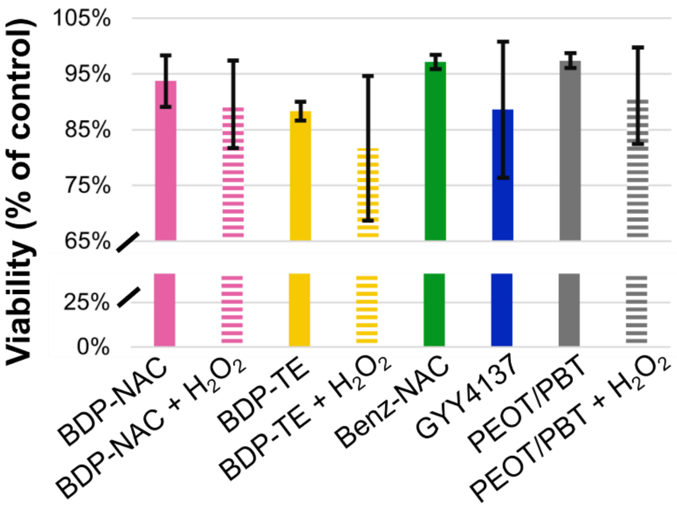

Fig S25: Viability assay of HMVECs on electrospun fiber mats. n = 5
